# The *Klf7/Glut2/Ins2* Axis Regulates Glucose Homeostasis

**DOI:** 10.3390/ijms27198553

**Published:** 2026-09-24

**Authors:** Chaofan Yang, Hebing Liu, Cao Wang, Fuxing Zhu, Mingyue Guo, Weiming Tian

**Affiliations:** School of Life Science and Technology, Harbin Institute of Technology, Harbin 150080, China

**Keywords:** glucose homeostasis, *Klf7*, pancreatic β-cell, *Glut2*, insulin

## Abstract

Krüppel-like factor 7 (*KLF7*) is a Cys_2_/His_2_ zinc-finger transcription factor implicated in metabolic regulation. Although *KLF7* variants are associated with type 2 diabetes (T2D), its role in β-cell glucose homeostasis remains unclear. *Klf7* is downregulated in β-cells from mice and humans with established diabetes, and high glucose directly suppresses *Klf7* expression in isolated islets. β-cell-specific *Klf7* knockout (β-KLF7KO) and overexpression (β-KLF7OE) mouse models were generated, along with AAV-mediated rescue models. Islet transcriptomics, glucose-stimulated insulin secretion assays, luciferase reporter assays, and lentiviral knockdown/overexpression in INS1 and 1.1B4 cells were performed. Here we show that *KLF7* is downregulated in β-cells from diabetic human donors and mice. β-cell-specific deletion of *Klf7* in mice impaired insulin secretion and glucose tolerance, accompanied by a 47% reduction in *Slc2a2* (*Glut2*) expression. *Klf7* overexpression in vitro suppressed insulin secretion, revealing opposing transcriptional regulation of *Glut2* and *Ins2*. Luciferase reporter assays confirmed that *Klf7* directly activates the *Glut2* promoter and represses the *Ins2* promoter, establishing an opposing transcriptional regulatory mechanism. This regulatory mechanism is conserved in the 1.1B4 human pancreatic islet fusion cell line, in which *KLF7* knockdown derepressed *INS* and suppressed *GLUT2* expression. AAV-mediated restoration of either *Klf7* or *Glut2* in β-KLF7KO mice largely rescued the metabolic defects, identifying *Glut2* as a principal downstream mediator. Given that *KLF7* declines under hyperglycaemic stress and its loss is sufficient to impair β-cell function, the *Klf7/Glut2/Ins2* axis may represent an early, targetable step in β-cell dysfunction; whether *KLF7* decline precedes the onset of glucose intolerance in a natural progression model remains to be determined by longitudinal studies.

## 1. Introduction

Glucose homeostasis maintains blood glucose levels within a narrow physiological range and depends on the coordinated interaction between insulin secretion and systemic tissue sensitivity [1,2,3]. Dysregulation of glucose homeostasis is a pivotal factor in the development and progression of diabetes mellitus, in which pancreatic β-cell dysfunction and insulin resistance converge to drive persistent hyperglycaemia [4,5,6]. Diabetes mellitus can thus be viewed as the clinical end point of failed glucose homeostasis; dissecting the β-cell-intrinsic machinery that maintains this homeostasis is central to understanding how the transition from normoglycaemia to T2D begins. In recent years, Krüppel-like factors (KLFs) have emerged as critical regulators of glucose homeostasis and β-cell function [7].

The KLFs are a family of Cys_2_/His_2_ zinc-finger transcription factors that serve as crucial regulators of physiological homeostasis [8]. Dysregulation of KLF family members is associated with metabolic diseases, including diabetes [7,9]. KLFs regulate the ATP/ADP ratio, insulin granule exocytosis, and insulin synthesis to maintain glucose homeostasis [7]. Well-characterised examples include *KLF6*, *KLF10*, *KLF11*, *KLF14*, and *KLF15* [10,11,12,13,14,15,16]. Among these, *KLF7* has emerged as a particularly important regulator, yet its role in β-cells remains poorly defined.

Accumulating evidence from our laboratory and others has established *KLF7* as a central regulator in diverse physiological and pathological processes, underscoring its broad functional significance [17]. Our prior work has demonstrated that *KLF7* modulates cardiac metabolic remodelling during hypertrophy through the *KLF7/PFKL/ACADL* axis [18], and plays a critical role in maintaining the balance of mitochondrial fission and fusion during myocardial infarction [19]. Furthermore, *KLF7* has been identified as a causal gene in autism spectrum disorder [20], and its deficiency disrupts circadian rhythm-related genes, triggering rhythm oscillations [21]. However, whether *KLF7* functions in pancreatic β-cells to regulate glucose homeostasis remains unknown, despite genetic links between *KLF7* variants and T2D risk.

*KLF7* overexpression in pancreatic β-cell lines suppresses glucose-stimulated insulin secretion (GSIS) [22], and human genetic studies have linked *KLF7* variants to type 2 diabetes risk [23,24]. Corroborating these genetic and functional links, transcriptomic analysis of laser-capture microdissected β-cells from type 2 diabetic donors (GSE20966) revealed that *SLC2A2 (GLUT2)* was the most profoundly downregulated β-cell functional gene, and this pattern was conserved in db/db mouse islets (GSE107489), where *Glut2* expression was reduced. Because glucose uptake, mediated primarily by the glucose transporter GLUT2, is the critical first step that triggers GSIS [25], we hypothesised that *KLF7* regulates β-cell function by controlling the expression of *Glut2* and insulin genes. We therefore asked whether *KLF7* is part of the β-cell-intrinsic machinery that maintains glucose homeostasis, and whether loss of *Klf7* is sufficient, on its own, to impair β-cell function and glucose tolerance.

To test this hypothesis, we generated pancreatic-specific *Klf7* knockout (KLF7KO), β-cell-specific *Klf7* knockout (β-KLF7KO), and β-cell-specific *Klf7* overexpression (β-KLF7OE) mouse models. We assessed glucose homeostasis and insulin secretion in these mice, performed transcriptomic profiling of isolated islets, and conducted AAV-mediated rescue experiments in which *Klf7* or *Glut2* was restored in β-KLF7KO mice. Transcriptional regulation of *Glut2* and *Ins2* by *Klf7* was examined through luciferase reporter assays and loss- and gain-of-function experiments in pancreatic β-cell lines. Together, these complementary approaches were designed to test whether *Klf7* couples glucose sensing to insulin synthesis in pancreatic β-cells.

## 2. Results

### 2.1. High Glucose Downregulates KLF7 Expression

To determine whether *KLF7* expression is altered in pancreatic β-cells under diabetic conditions, we first examined transcriptomic datasets from human type 2 diabetic donors and four independent mouse diet-induced metabolic-stress datasets. In laser-capture microdissected (LCM) β-cells from T2D donors (GSE20966), *KLF7* expression was significantly reduced by 22.2% compared with non-diabetic controls (Figure 1A). The two groups (10 donors each) were comparable in sex distribution and BMI, but the T2D donors were older (67 ± 7 vs. 60 ± 5 years; *p* = 0.019), and fasting glucose, HbA1c, insulin and HOMA-derived indices were not reported in the original study; donor-level information was therefore insufficient for a full multivariable adjustment, and this comparison is unadjusted (Table S1). Across four independent mouse diabetic transcriptomic datasets (GSE107489 db/db islets, GSE224797 ob/ob β-cells, GSE330587 HFD islets, and GSE294866 HFD β-cells), *Klf7* was downregulated. When normalised to respective wild-type (WT), pooled *Klf7* expression in diabetic samples was decreased by 21.2% (Figure 1B). Across four independent mouse datasets, two overt diabetes models (GSE107489, db/db islets; GSE224797, ob/ob β-cells) and two diet-induced metabolic-stress models, one of which additionally carries a human T2D risk variant (GSE330587, HFD-fed Pax4-R192H knock-in mice vs. Pax4 WT littermates on the same diet; GSE294866, β-cells from HFD-fed mice), *Klf7* was downregulated, and the direction of change was consistent in every dataset (per-dataset effect sizes are given in Table S2). Because these datasets differ in platform, library preparation and genetic background, no cross-dataset batch correction was applied; instead, each dataset was normalised to its own wild-type control (WT = 1.0) before pooling, so that the pooled comparison reflects within-dataset fold changes rather than platform or baseline differences. Within each dataset, *Klf7* was lower in the diabetic group than in the corresponding control (GSE107489, log_2_FC = −0.46, n = 3 vs. 3; GSE224797, log_2_FC = −0.58, n = 5 vs. 10; GSE330587, log_2_FC = −0.40, n = 3 vs. 3; GSE294866, log_2_FC = −0.04, n = 4 vs. 3), so the direction of change is consistent across all four datasets.

Receiver operating characteristic (ROC) analysis showed that *KLF7*/*Klf7* expression discriminated diabetic from non-diabetic β-cells across species, with moderate accuracy in humans and strong discrimination in mice (Figure 1C). Taken together, these results demonstrate that *KLF7* is downregulated in diabetic β-cells across three classical mouse models of diabetes (db/db, ob/ob, and HFD) as well as in type 2 diabetic donors, indicating a conserved, cross-species pattern of *KLF7* downregulation in diabetes. These findings establish an association between diabetes and reduced KLF7 expression; whether *Klf7* loss is sufficient on its own to impair β-cell function was tested directly in the genetic and viral models described below.

We next asked whether the decrease in *KLF7* was selective among the *KLF* family. Analysis of all detectable *KLFs* in human LCM β-cells (GSE20966) revealed that *KLF7* was the only member showing a statistically significant change in T2D (Figure S1A). In mouse islets, 3 of 15 *Klf* genes were significantly downregulated in db/db compared with WT mice; among these, *Klf7* showed a consistent cross-species reduction (GSE107489) (Figure S1B). *KLF7* showed the most pronounced and consistent cross-species downregulation among *KLF* family members. To explore the functional context of *KLF7* loss, we examined its correlation with 9 key β-cell functional genes in human LCM β-cells. *KLF7* expression was positively correlated with *GLUT2*, *ABCC8*, *MAFA*, *PDX1*, *NKX6-1*, *NEUROD1*, *INS*, *KCNJ11* and *GCK* (Figure S1C). A gradient heatmap across WT, db/+ and db/db mouse islets further revealed coordinated downregulation of *Klf7* together with 9 β-cell functional genes (*Glut2*, *Gck*, *Pdx1*, *Mafa*, *Neurod1*, *Ins2*, *Kcnj11*, *Nkx6-1*, *Abcc8*) (Figure S1D). These findings suggest that *Klf7* loss occurs within a broader programme of β-cell gene repression and is closely associated with the expression of genes essential for glucose uptake and insulin secretion. Having confirmed KLF7 reduction in end-stage disease, we asked whether this represents a downstream consequence of hyperglycaemia itself.

We exposed isolated mouse islets to high glucose (25 mM) for 48 h. RT-PCR analysis showed that *Klf7* mRNA levels were significantly reduced in high-glucose-treated islets compared with low glucose (5.5 mM) controls (Figure 1D). This decrease was corroborated at the protein level: KLF7 protein abundance, assessed by Western blot, was also significantly reduced under high-glucose conditions (Figure 1E,F). These results indicate that hyperglycaemia is sufficient to suppress KLF7 expression in pancreatic islets at both the transcript and protein levels.

Having established that *Klf7* is downregulated under diabetic conditions and that high glucose directly suppresses its expression, we next investigated whether *Klf7* loss causally contributes to β-cell dysfunction.

### 2.2. β-Cell-Specific Klf7 Deletion and Overexpression Differentially Impact Glucose Tolerance and Insulin Secretion

To establish the causal contribution of *Klf7* downregulation to β-cell dysfunction, we generated β-cell-specific Klf7 knockout mice by crossing *Klf7*^fl/fl^ mice with RIP-Cre transgenic mice (β-KLF7KO). *Klf7* mRNA levels in isolated islets were reduced by 78.3% compared with *Klf7*^fl/fl^ littermates (Figure 2A), and KLF7 protein was correspondingly reduced (Figure 2B). β-KLF7KO mice were born at expected Mendelian ratios and exhibited body weights comparable to controls from 6 to 18 weeks of age (Figure 2C), indicating that *Klf7* is dispensable for embryonic survival and gross postnatal development.

Despite normal body weight, β-KLF7KO mice displayed significantly elevated fasting blood glucose at 12 weeks of age (Figure 2D) and reduced fasting plasma insulin (Figure 2E). Intraperitoneal glucose tolerance tests (GTT, 1.5 g/kg) revealed markedly impaired glucose clearance in β-KLF7KO mice, with blood glucose remaining significantly elevated throughout the time course (Figure 2F). The area under the curve (AUC) was significantly increased compared with controls (Figure 2G).

In contrast, under our experimental conditions no detectable difference in insulin sensitivity was observed between genotypes, as assessed by insulin tolerance tests (ITT, 0.75 U/kg); the ITT AUC was not significantly different and the genotype × time interaction was not significant (Figure 2H,I). The dissociation between impaired glucose tolerance and an unaltered insulin-sensitivity readout is consistent with a metabolic phenotype that arises from defective insulin secretion rather than systemic insulin resistance.

We next examined pancreatic *Klf7* knockout mice driven by Pdx1-Cre (KLF7KO), in which Klf7 is deleted throughout the pancreatic epithelium. *Klf7* mRNA levels were reduced by 48.0% in KLF7KO islets (Figure S2A), with corresponding loss of KLF7 protein in the pancreas (Figure S2B). KLF7KO mice exhibited normal body weight (Figure S2C) but displayed elevated fasting blood glucose (Figure S2D), reduced fasting plasma insulin (Figure S2E), and impaired glucose tolerance (Figure S2F,G). However, unlike β-KLF7KO mice, KLF7KO mice additionally exhibited significantly reduced insulin sensitivity (Figure S2H,I), consistent with *Klf7* deletion in extra-β-cell pancreatic lineages. Notably, RNA-seq analysis of isolated islets revealed no compensatory upregulation of any other *Klf* family member in either β-KLF7KO or KLF7KO mice after multiple-testing correction (Figure S3A,B), and islet morphology was unremarkable as assessed by haematoxylin and eosin staining (Figure S3C–F). These findings indicate that *Klf7* functions non-redundantly in β-cells and that its deletion impairs glucose homeostasis without triggering compensatory transcriptional responses or altering islet architecture.

Having established that *Klf7* deficiency impairs β-cell function, we next investigated whether *Klf7* overexpression would produce opposing effects. We established β-cell-specific *Klf7* overexpression (β-KLF7OE) in WT mice via pancreatic ductal injection of AAV8-*Ins2-Klf7*, resulting in a 2.4-fold increase in *Klf7* mRNA in isolated islets compared with WT controls (Figure 3A). KLF7 protein was correspondingly increased (Figure 3B). β-KLF7OE mice exhibited fasting blood glucose comparable to that of WT controls (Figure 3C), but showed significantly reduced fasting plasma insulin (Figure 3D).

Intraperitoneal GTT (1.5 g/kg) revealed that β-KLF7OE mice had improved glucose clearance compared with WT controls (Figure 3E). The GTT AUC was significantly reduced (Figure 3F). ITT (0.75 U/kg) revealed no significant difference in insulin sensitivity between β-KLF7OE and WT mice (Figure 3G,H). Taken together, these results demonstrate that *Klf7* overexpression in β-cells produces divergent metabolic effects. Although fasting insulin is reduced, glucose tolerance improves, and systemic insulin sensitivity shows no detectable change under our experimental conditions (Figure 3G,H), a pattern also observed in β-KLF7KO mice. Given the directions of secretory imbalance in the two models, we profiled the islet transcriptome of β-KLF7KO mice and dissected the underlying regulatory mechanism.

### 2.3. Cellular Pathways Involved in Insulin Secretion Are Impaired in β-KLF7KO Islets

To elucidate the molecular mechanisms underlying the metabolic phenotype of β-KLF7KO mice, we performed an exploratory RNA-seq analysis on islets isolated from WT and β-KLF7KO mice. Among 25,061 genes analysed, we identified 481 significantly upregulated and 180 significantly downregulated genes in β-KLF7KO islets using this discovery-oriented threshold (|log_2_FC| > 1.0, *p* < 0.05; n = 4 per group) (Figure 4A). GO enrichment analysis revealed that differentially expressed genes were significantly enriched in biological processes related to insulin secretion, glucose metabolism, transmembrane transport, and regulation of secretion (Figure 4B). KEGG pathway analysis further confirmed that the insulin secretion pathway was among the most prominently affected categories (Figure 4C).

Heatmap visualisation of key functional categories revealed coordinated transcriptional dysregulation in β-KLF7KO islets. Genes involved in secretion regulation, including *Pcsk1*, *Cltrn*, *Resp18*, and *Cadps*, were broadly altered. Notably, among genes mediating transmembrane transport, *Glut2*, which encodes the primary glucose transporter in rodent β-cells, was the most strongly downregulated transporter gene under our exploratory criterion. Genes central to glucose metabolism, including *Ins2* and *G6pc2*, were also markedly upregulated, and several potassium channel genes (*Kcnma1*, *Kcnj15*, *Kcna2*) were downregulated, consistent with impaired ion flux required for insulin exocytosis (Figure 4D).

We next examined whether the downregulation of *Glut2* was specific among the GLUT transporter family. Analysis of all detectable GLUT family members showed that *GLUT2* was the only GLUT gene significantly downregulated in human T2D β-cells (GSE20966) (Figure S4A), and *Glut2* was the most significantly reduced GLUT member in both *db/db* mouse islets (GSE107489) (Figure S4B) and β-KLF7KO islets (Figure S4C). All other GLUT family members remained largely unchanged across all three datasets. Collectively, these transcriptomic data reveal that *Klf7* deficiency extensively remodels the transcriptional landscape governing insulin secretion and glucose metabolism pathways in β-cells. Notably, *Glut2* was markedly downregulated, whereas *Ins2* was upregulated, an opposing expression pattern consistent with opposing regulatory buffer of *Klf7* at its direct transcriptional targets. Having identified *Glut2* downregulation as a key transcriptional consequence of *Klf7* loss, we next examined whether impaired glucose uptake underlies the insulin secretion defect in β-KLF7KO islets.

### 2.4. Klf7 Deficiency Impairs Glucose Uptake Through Glut2 Downregulation

To pinpoint the specific step in the insulin secretory pathway affected by *Klf7* deficiency, we assessed GSIS in isolated islets. Under basal conditions (2.8 mM glucose), insulin secretion was comparable between WT and β-KLF7KO islets (Figure 5A). However, upon stimulation with 16.7 mM glucose, β-KLF7KO islets exhibited significantly impaired insulin secretion compared with WT controls. By contrast, depolarisation with 30 mM KCl elicited robust insulin secretion from β-KLF7KO islets that exceeded WT levels, indicating that the exocytotic machinery remains functionally intact. To quantify the secretory defect, we calculated the glucose stimulation index (SI = insulin at 16.7 mM glucose/insulin at 2.8 mM glucose). β-KLF7KO islets showed a 2.6-fold reduction in glucose-stimulated SI compared with WT (Figure 5B). In contrast, the KCl stimulation index was comparable between genotypes (Figure 5C). These results demonstrate that *Klf7* deficiency selectively impairs glucose-stimulated insulin secretion while sparing the distal exocytotic machinery, pointing to a defect upstream of membrane depolarisation, most likely at the level of glucose sensing or uptake.

Consistent with this interpretation, *Glut2* expression was markedly reduced in β-KLF7KO islets at the mRNA level (Figure 5D). GLUT2 protein was correspondingly decreased by approximately 47% in β-KLF7KO islets compared with WT controls (Figure 5E). To directly assess the functional consequence of reduced GLUT2 expression, we measured glucose uptake in INS1 β-cells after *Klf7* knockdown, using the fluorescent glucose analogue 2-NBDG. *Klf7* knockdown significantly reduced 2-NBDG uptake by 48.7% compared with control cells (Figure 5F).

We next examined whether *Klf7* expression correlated specifically with *Glut2* among the *Gluts*. In human pancreatic islets (GSE20966), *KLF7* expression showed a positive correlation with *GLUT2* (*r* = 0.426, *p* = 0.061) (Figure S5A), but not with *GLUT1* (Figure S5B) or *GLUT3* (Figure S5C). In mouse islets (GSE107489), *Klf7* expression was strongly and significantly correlated with *Glut2* (Figure S5D). *Klf7* was negatively correlated with *Glut1* (Figure S5E) and only weakly correlated with *Glut3* (Figure S5F). The selective and cross-species correlation between *KLF7/Klf7* and *GLUT2/Glut2*, but not other GLUT family members, is consistent with the model that *KLF7* specifically regulates *Glut2* expression in β-cells. However, because human β-cells sense glucose predominantly through *GLUT1* and *GLUT3*, whether *KLF7* directly modulates human *GLUT1/GLUT3* expression remains to be tested; this is discussed further in the Discussion Section.

Taken together, these data indicate that *Klf7* is required for normal *Glut2* expression and glucose uptake in β-cells, and that this defect precedes the impairment in GSIS. Having established that *Klf7* deficiency impairs *Glut2* expression and glucose uptake, we next investigated whether *Klf7* directly regulates the *Glut2* and *Ins2* genes at the transcriptional level.

### 2.5. Klf7 Directly Targets Glut2 and Ins2 in β-Cells

Our transcriptomic and functional analyses consistently identified *Glut2* and the insulin genes as key downstream effectors of *Klf7*. To determine whether *Klf7* might directly regulate these genes, we first performed bioinformatic analysis of *KLF7* binding sites across their promoters. Promoter scanning (−3000 to +500 bp relative to TSS) revealed multiple CACCC and GGGTG KLF-binding motifs in the mouse and human *Glut2/SLC2A2* promoters (7 and 13 sites, respectively) (Figure S6A,B). In marked contrast, the human *INS* promoter displayed a striking enrichment of 58 *KLF*-binding motifs, including a CCACCC box at −82 bp within the core promoter, whereas the mouse *Ins2* promoter contained 3 such motifs (Figure S6C,D). Cross-database validation supported *Glut2/SLC2A2* and *Ins2/INS* as predicted *Klf7* targets across multiple independent algorithms (Figure S6E). These predictions provided the structural rationale to experimentally test whether *Klf7* directly regulates these genes.

We performed loss- and gain-of-function experiments in rat INS1 cells. Lentiviral knockdown of *Klf7* (sh-*Klf7*) significantly reduced *Klf7* mRNA and produced opposing effects on its target genes, with *Ins1* and *Ins2* markedly upregulated and *Glut2* significantly downregulated (Figure 6A). Despite the increase in insulin gene transcription, total insulin secretion from *Klf7*-knockdown INS1 cells was modestly reduced by approximately 13% compared with control cells (Figure 6B), revealing a dissociation between insulin mRNA levels and insulin secretion that is consistent with the concurrent loss of *Glut2*-mediated glucose sensing. Conversely, lentiviral overexpression of *Klf7* (lenti-*Klf7*) in INS1 cells suppressed *Ins1* and *Ins2* expression while upregulating *Glut2* (Figure 6C). Total insulin secretion was reduced in *Klf7*-overexpressing cells (Figure 6D), consistent with reduced insulin gene expression. Taken together, these results demonstrate that *Klf7* exerts opposing transcriptional effects on *Glut2* and *Ins2*, establishing an opposing regulatory buffer in which *Klf7* reciprocally controls glucose uptake and insulin production.

To assess whether this regulatory relationship extends to human β-cells, we performed parallel experiments in the 1.1B4 human pancreatic islet fusion cell line, a human β-cell-origin model amenable to molecular genetic manipulation that has been employed for transcriptional and signalling studies in human β-cell biology. *KLF7* knockdown (sh-*KLF7*) significantly upregulated *INS* and downregulated *GLUT2* (Figure 6E), whereas *KLF7* overexpression suppressed *INS* and increased *GLUT2* (Figure 6F). These results provide preliminary evidence that the *KLF7/GLUT2/INS* regulatory logic is conserved in a human β-cell model, although the hybrid 1.1B4 line does not fully recapitulate native human β-cell biology and confirmation in primary human islets or stem cell-derived β-cells is required. To determine whether *Klf7* directly binds the *Glut2* and *Ins2* promoters, we performed luciferase reporter assays in 293T cells. *Klf7* significantly repressed *Ins2* promoter-driven luciferase activity (Figure 6G) and activated the *Glut2* promoter (Figure 6H). These results demonstrate that *Klf7* directly regulates the *Glut2* and *Ins2* promoters. Having established that *Klf7* directly regulates *Glut2* and *Ins2* transcription, we next investigated whether restoring *Klf7* expression in β-KLF7KO mice could reverse the metabolic defects caused by *Klf7* deficiency.

### 2.6. Restoring the Klf7/Glut2/Ins2 Axis Rescues Glucose Homeostasis in β-KLF7KO Mice

To determine whether the metabolic defects caused by *Klf7* deficiency are reversible, we performed rescue experiments by restoring *Klf7* expression in β-KLF7KO mice. β-KLF7KO mice were injected via the pancreatic duct with AAV8-*Ins2*-*Klf7* (hereafter referred to as *Klf7* rescue), which restored KLF7 protein in islets to levels comparable to WT controls (Figure 7A,B) and *Ins2* mRNA in islets to levels comparable to WT controls (Figure 7C). *Klf7* rescue significantly reduced fasting blood glucose compared with β-KLF7KO mice (Figure 7D) and restored fasting plasma insulin to near-WT levels (Figure 7E). Intraperitoneal GTT (1.5 g/kg) demonstrated that *Klf7* rescue significantly improved glucose clearance (Figure 7F), with the GTT AUC reduced from β-KLF7KO levels (Figure 7G). The improved ITT (0.75 U/kg) after *Klf7* rescue (Figure 7H,I) is most consistent with improved whole-body glucose handling secondary to the normalisation of glycaemia, rather than with a direct increase in systemic insulin sensitivity. Taken together, these results demonstrate that restoring KLF7 expression is sufficient to largely normalise glucose homeostasis in β-KLF7KO mice, confirming that *Klf7* deficiency causally underlies the metabolic phenotype.

We next asked whether directly targeting the key transcriptional target *Glut2* would be sufficient to achieve the same effect. We delivered AAV8-*Ins2-Glut2* to β-KLF7KO mice (hereafter referred to as *Glut2* rescue). This intervention restored GLUT2 protein to approximately 2.3-fold the β-KLF7KO levels (Figure 8A,B). Importantly, *Ins2* mRNA levels remained unchanged in *Glut2* rescue islets compared with β-KLF7KO controls (Figure 8C), confirming that *Glut2* rescue acts downstream of *Klf7* without altering *Ins2* transcription. *Glut2* rescue normalised fasting blood glucose (Figure 8D) and restored fasting plasma insulin to levels that exceeded WT (Figure 8E), consistent with supranormal glucose uptake driving enhanced insulin secretion. GTT and ITT analyses confirmed that *Glut2* rescue significantly improved glucose tolerance (Figure 8F,G) and improved the ITT response (Figure 8H,I), the latter reflecting a secondary consequence of glycaemic normalisation rather than a direct increase in systemic insulin sensitivity.

These results show that restoring *Glut2* alone rescues glucose homeostasis in β-KLF7KO mice, confirming that *Glut2* downregulation is the functionally limiting consequence of *Klf7* loss. At the same time, the supranormal insulin secretion that accompanies *Glut2* rescue in the continued absence of *Klf7* reveals that *Ins2* repression by *Klf7* normally restrains insulin secretion. The *Klf7/Glut2/Ins2* axis is therefore required for normal β-cell function.

## 3. Discussion

The pancreatic β-cell calibrates its insulin secretion to match fluctuating blood glucose levels, a process that depends on the coordinated transcription of genes governing glucose uptake and insulin biosynthesis [3,26]. This study identifies *Klf7* as a direct transcriptional regulator of both glucose uptake and insulin gene expression in pancreatic β-cells. *Klf7* directly activates *Glut2*, the predominant glucose transporter in rodent β-cells [27], while repressing *Ins2*, the principal rodent insulin gene. This opposing regulatory mechanism is concentration-sensitive as both *Klf7* deficiency and excess disrupt β-cell function in vivo and in vitro. These observations suggest that β-cell function depends on *Klf7* expression being maintained within a permissive range, with both deficiency and excess detrimental at the cell-autonomous level. These findings define the *Klf7/Glut2/Ins2* axis as a transcriptional mechanism that couples glucose sensing to insulin secretion in pancreatic β-cells.

The rescue experiments provide further insight into the functional hierarchy of the *Klf7/Glut2/Ins2* axis. Restoring *Glut2* expression alone in β-KLF7KO mice, without correcting the underlying *Klf7* deficiency, was sufficient to normalise fasting blood glucose and reverse the defects in glucose tolerance and insulin levels. Notably, *Ins2* mRNA levels remained unchanged in *Glut2* rescue islets, indicating that the therapeutic benefit does not require correction of *Ins2* expression and is instead mediated through *Glut2*-dependent restoration of glucose sensing and uptake. Moreover, plasma insulin levels in *Glut2* rescue mice exceeded those of controls, indicating that restoring glucose uptake without reinstating *Ins2* repression drives insulin secretion beyond physiological levels. Both arms of the *Klf7/Glut2/Ins2* axis are therefore necessary for homeostatic control [28].

The supranormal insulin secretion observed in *Glut2* rescue mice is the expected outcome of restoring glucose uptake while *Ins2* remains derepressed in the continued absence of *Klf7*. When *Glut2* is exogenously restored, glucose-stimulated secretory drive is reinstated without the transcriptional constraint on insulin production normally imposed by *Klf7*, yielding a system in which both glucose uptake and insulin biosynthesis are concurrently elevated. This unmasks the physiological function of *Klf7*-mediated *Ins2* repression, which limits insulin secretion when glucose sensing is intact and prevents secretory overshoot. The *Klf7/Glut2/Ins2* axis thus operates through an opposing regulatory mechanism, in which *Klf7* concurrently activates *Glut2* to promote glucose uptake while repressing *Ins2* to restrain insulin production. Whether chronic loss of this brake eventually leads to β-cell exhaustion through ER stress or metabolic overload remains an important open question for long-term follow-up studies.

In INS1 cells subjected to *Klf7* knockdown, *Ins1* and *Ins2* transcription were markedly upregulated, yet total insulin secretion was reduced. *Klf7* overexpression inhibited *Ins1* and *Ins2* expression, yet total insulin secretion was reduced as well. In the overexpression setting, the secretory deficit is attributable to suppressed insulin gene transcription, consistent with *Klf7*-mediated *Ins2* repression. In the knockdown setting, however, the dissociation between elevated insulin mRNA and reduced secretion is less straightforward, and three non-mutually exclusive mechanisms have been considered. First, the concurrent loss of *Glut2* expression in *Klf7*-knockdown cells may reduce glucose uptake to an extent that ATP production falls below the threshold required to trigger secretion, even though insulin gene transcription is elevated [29,30]. Second, the marked increase in insulin mRNA could in principle elevate the biosynthetic load on the endoplasmic reticulum and trigger ER stress with compensatory attenuation of translation [31,32]. However, our islet RNA-seq revealed no elevation of classical ER-stress markers (*BIP* and *GRP94* were instead decreased, and *CHOP* was unchanged), arguing against ER-stress-mediated translational suppression at this time point. Third, *Klf7* may indirectly influence the expression of genes involved in insulin granule maturation or trafficking, thereby dissociating insulin transcription from secretion [33]. These opposing outcomes reinforce the core logic of the *Klf7/Glut2/Ins2* axis. When both regulatory arms are disrupted concurrently, the deficit in one arm determines the secretory outcome. In *Klf7* deficiency, *Glut2* downregulation limits secretion despite elevated insulin mRNA. In *Klf7* excess, *Ins2* suppression restricts insulin production despite enhanced glucose uptake. This interdependence demonstrates that *Klf7* calibrates insulin secretion by governing glucose uptake and insulin gene expression in tandem, and that neither regulatory arm is dispensable for normal β-cell function. This assessment is based on whole-islet transcripts at a single time point, so an ER stress response confined to β-cells, or one developing in chronic or end-stage disease, would not necessarily be captured. In addition, activation of some unfolded protein response branches occurs through protein phosphorylation or *XBP1* splicing and need not be reflected in total transcript levels.

The opposing regulatory mechanism described above implies that *Klf7* expression must be maintained within a permissive range for β-cells to function normally, with both deficiency and excess being detrimental. The evidence for this is strongest at the cell-autonomous level. In INS1 cells, both *Klf7* knockdown and overexpression impaired insulin secretion, consistent with an opposing regulatory mechanism. In vivo, however, the picture is more nuanced. β-KLF7OE mice, in which *Klf7* was overexpressed approximately 2.4-fold via AAV delivery [34], exhibited improved glucose tolerance (Figure 3E,F) but no detectable change in systemic insulin sensitivity (Figure 3G,H), despite a significant reduction in fasting plasma insulin (Figure 3D). It should be noted that insulin sensitivity in this study was assessed by the insulin tolerance test and the interpretation is confined to our experimental conditions. A similar absence of a detectable difference was observed in the β-KLF7KO, whereas the altered insulin tolerance responses in the KLF7KO and in the rescue settings may involve indirect or non-β-cell effects; these should not be taken as direct evidence of a change in systemic insulin sensitivity. Rather than reflecting a single uniform defect, this dissociation is compatible with the opposing regulation of *Glut2* and *Ins2* by *Klf7*, in which enhanced glucose sensing and disposal are offset at the level of insulin secretion; the net outcome is improved glucose tolerance in the absence of altered systemic insulin sensitivity. The increase in islet *Klf7* mRNA achieved by AAV delivery may lie within the permissive range, particularly given the mosaic nature of AAV-mediated pancreatic transduction, although transduction efficiency was not directly quantified [34,35,36]. We propose that this concentration-sensitive regulatory logic is a cell-intrinsic property of β-cells, and that its in vivo manifestation can be shaped by variables such as the magnitude and distribution of transgene expression. Whether higher levels of *Klf7* overexpression would eventually impair glucose tolerance in vivo remains an open question.

It is also worth considering how peripheral tissues decode a reduced β-cell insulin signal. Insulin action in these tissues is not a simple linear function of plasma insulin but can exhibit threshold-dependent, bistable response dynamics, in which a graded change in insulin elicits an abrupt switch in tissue glucose disposal [37]. Within this framework, even a modest reduction in β-cell insulin secretion may suffice to push muscle glucose disposal below its switch-on threshold, converting a small secretory deficit into a disproportionately large effect on whole-body glucose clearance. This may explain why a contained molecular defect in β-cells, the loss of *Klf7*-driven *Glut2* expression, produces such a pronounced hyperglycaemic phenotype in vivo.

The improved insulin tolerance observed after *Klf7* or *Glut2* rescue in β-KLF7KO mice should likewise not be interpreted as an increase in systemic insulin sensitivity. The insulin tolerance test measures how far a fixed dose of exogenous insulin lowers blood glucose, and when fasting glucose is markedly elevated, as in untreated β-KLF7KO mice, the same dose acts against a larger glucose pool, so that the measured fall in blood glucose is diluted. Once rescue restores fasting glucose towards normal, the same dose produces a larger relative fall and the insulin tolerance test AUC improves accordingly. Because both rescue vectors are delivered selectively to β-cells (AAV8-*Ins2-Klf7* and AAV8-*Ins2-Glut2*), a direct action of the transgene on peripheral tissues is mechanistically implausible; differences in experimental timing between the baseline and rescue cohorts may also contribute. The improvement in insulin tolerance after rescue is therefore best explained as a secondary consequence of the normalisation of glycaemia.

Beyond its concentration-sensitive regulation, the selectivity of *Klf7* among KLFs warrants consideration [10]. Among the seventeen KLFs, *Klf7* exhibited the most pronounced and consistent downregulation across human and mouse diabetic β-cell datasets, and neither β-KLF7KO nor KLF7KO islets showed compensatory upregulation of any other *Klf* gene. These observations point to a non-redundant, isoform-specific role for *Klf7* in the β-cell. How hyperglycaemia suppresses *Klf7* in the first place remains an open question. Given the rapid decline of both *Klf7* mRNA and protein upon high-glucose exposure, several non-exclusive mechanisms may operate at transcriptional and post-transcriptional levels, including *ChREBP*-dependent promoter repression, miR-375-mediated mRNA degradation, and oxidative-stress-driven proteasomal depletion of KLF7 protein [31,38,39,40,41]. Determining which of these predominates in vivo, and whether *Klf7* decline indeed precedes glucose intolerance in a natural progression model, will require longitudinal and mechanistic studies. It should be emphasised that the reduced *KLF7* expression documented across human and mouse datasets is association evidence, whereas the conclusion that *KLF7* directly regulates β-cell insulin secretion rests on the functional experiments performed in this study. Considered together, these data indicate that *Klf7* dysregulation is one contributing factor to β-cell dysfunction and to impaired glucose-stimulated insulin secretion, rather than a universal primary driver of diabetes.

Translating these findings to human biology must contend with a well-documented species difference [42,43]. Rodent β-cells depend almost exclusively on *Glut2* for glucose uptake, and our data establish *Klf7* as a direct transcriptional driver of this transporter [27]. Human β-cells, by contrast, rely primarily on *GLUT1* and *GLUT3*, with *GLUT2* expressed at substantially lower levels [44,45,46,47]. Despite this divergence, the clinical importance of *GLUT2* in human glucose homeostasis is underscored by the finding that biallelic loss-of-function mutations in *GLUT2* cause Fanconi–Bickel syndrome, a glycogen storage disorder characterised by impaired glucose tolerance, postprandial hyperglycaemia, and fasting hypoglycaemia [48], and can present as transient neonatal diabetes prior to the emergence of the full syndromic phenotype [49]. Nevertheless, whether the *KLF7/GLUT*2 axis carries equivalent functional weight in the human islet, where *GLUT2* is expressed at substantially lower levels than in rodents, remains uncertain. Our experiments in the 1.1B4 human pancreatic islet fusion cell line provide preliminary evidence that the regulatory logic is conserved [50]. *KLF7* knockdown in these cells derepressed *INS* and suppressed *GLUT2*, recapitulating the pattern seen in rodent lines. While 1.1B4 cells are a hybrid line that may not fully recapitulate the molecular physiology of native human β-cells, determining whether *KLF7* also regulates the dominant human β-cell glucose transporters *GLUT1* and *GLUT3*, in addition to *INS*, will establish how broadly this opposing regulatory mechanism applies to human β-cell biology.

Several limitations of this study warrant consideration. β-KLF7KO mice carry the RIP-Cre driver, which contains a human growth hormone (hGH) minigene that is expressed in islets and can affect β-cells independently of the floxed allele [51]. Cre-negative *Klf7^fl/fl^* littermates were used as controls instead of a Cre-only cohort, and we regard this as a limitation. This is unlikely to drive the phenotype we report: restoring *Klf7* or *Glut2* within the same Cre-positive background largely normalised glucose homeostasis (Figure 7 and Figure 8), and in comparable β-cell-specific knockout studies using the same driver, RIP-Cre^+/−^ and *Klf7^fl/fl^* littermates showed similar body weight and blood glucose, which is why *Klf7^fl/fl^* littermates served as controls [52,53]. A Cre-only cohort nevertheless remains the most rigorous control and will be included in future work. All metabolic phenotyping in the present study was conducted solely in male mice, and a confirmatory female cohort was not included. Given the well-documented sex differences in β-cell biology [54,55,56], a dedicated cohort in females is warranted and will be addressed in future studies to determine whether the *Klf7/Glut2/Ins2* axis operates similarly in females. Our human validation was limited to the 1.1B4 pancreatic islet fusion cell line [50]. While these experiments demonstrated conservation of the regulatory logic, confirmation in primary human islets or stem cell-derived β-cells would substantially strengthen the translational argument. The signalling pathways that link hyperglycaemia to *Klf7* downregulation remain to be mapped, and the structural determinants of promoter selectivity, particularly the marked difference in *KLF7* binding-site density between the human *INS* and mouse *Ins2* promoters, await deeper biochemical analysis. Insulin sensitivity was assessed by the insulin tolerance test, which has limited power to detect modest insulin-resistance phenotypes; a hyperinsulinaemic–euglycaemic clamp would be required to address this question more conclusively.

Despite these limitations, this study establishes the *Klf7/Glut2/Ins2* axis as a transcriptional mechanism that couples glucose sensing to insulin secretion in β-cells. Disruption of this opposing regulatory link, whether through *Klf7* loss or excess, is sufficient to disturb β-cell function and may contribute to the loss of glucose competence that marks the progression from normoglycaemia to type 2 diabetes.

## 4. Materials and Methods

Gene nomenclature follows the conventions of the respective species throughout: mouse and rat gene symbols are italicised with an initial capital (e.g., *Klf7*, *Glut2*, *Ins1*, *Ins2*, *Gapdh*), human gene symbols are italicised and fully capitalised (e.g., *KLF7*, *GLUT2*, *INS*, *GAPDH*), and proteins are written in roman upper case (e.g., KLF7, GLUT2, GAPDH). Where a molecule is referred to in panels derived from different species, the symbol follows the species of the sample analysed in that panel.

### 4.1. Cell Culture

INS1 cells (supplied by the School of Life Science and Technology, Harbin Institute of Technology) were grown in RPMI 1640 supplemented with 10% FBS, 10 mM Hepes, 2 mM glutamine, 0.05 mM β-mercaptoethanol, 1 mM sodium pyruvate, and 1× penicillin–streptomycin solution. 1.1B4 cells (KCB 2015017, kmcellbank, Kunming, China) were cultured in RPMI 1640 supplemented with 10% FBS, and 1× penicillin–streptomycin solution. All cell cultures were maintained at 37 °C in a humidified atmosphere containing 5% CO_2_.

### 4.2. Animal Studies

Pdx1-Cre^+/−^ mice and *Klf7^fl/fl^* were purchased from Cyagen Co., Ltd. RIP-Cre^+/−^ mice were supplied by the School of Life Science and Technology, Harbin Institute of Technology. All mice were on a C57BL/6J background. β-cell-specific *Klf7* knockout (RIP-Cre;*Klf7^fl/fl^*) mice were generated by crossing RIP-Cre^+/−^ mice with *Klf7^fl/fl^* mice to obtain RIP-Cre; *Klf7^fl/wt^* mice, which were then crossed with *Klf7^fl/fl^* mice to yield RIP-Cre; *Klf7^fl/fl^* mice. Pancreatic *Klf7* knockout (Pdx1-Cre; *Klf7^fl/fl^*) mice were generated by the same breeding strategy, with Pdx1-Cre^+/−^ in place of RIP-Cre^+/−^ mice. Experimental mice carried the Cre transgene in heterozygosity, and Cre-negative *Klf7^fl/fl^* littermates from the same breeding colony were used as controls; this control strategy follows that of previous β-cell-specific knockout studies using the same RIP-Cre driver.

Male C57BL/6J mice (18–20 g, 6–8 weeks) were purchased from Changsheng Biotechnology Co., Ltd. Overexpression of *Klf7* in pancreatic β-cells was achieved utilising adeno-associated virus (AAV) technology. Specifically, pancreas-targeted adeno-associated virus serotype 8 (AAV8) was engineered to overexpress *Klf7* (AAV8-*Ins2-Klf7*), constructed and packaged by Shanghai GeneChem Co., Ltd. Similarly, the AAV8 technology was used to overexpress *Glut2* (AAV8-*Ins2-Glut2*) in β-cells. Animal experiments were carried out in strict accordance with the Guide for the Care and Use of Laboratory Animals of the National Institutes of Health. All mice were maintained on a 12 h light/dark cycle and fed ad libitum with standard chow and tap water at the Harbin Institute of Technology animal facility. All animal experiments were approved and performed in accordance with guidelines set forth by the Harbin Institute of Technology Institutional Animal Care and Use Committee (IACUC-2026008).

### 4.3. Viral Infection

To investigate the effects of *Klf7* knockdown (or overexpression), cellular models were subjected to lentiviral transduction. Following a 72 h incubation period to allow for transduction and gene expression modulation, RT-PCR (Table 1) and Western blot analyses were carried out to evaluate the efficiency of the knockdown (or overexpression). Relative expression was calculated using the 2^−ΔΔCt^ method and normalised to *Gapdh*.

**Table 1:** RT-PCR primer sequences.

Gene	Forward Primer (5′→3′)	Reverse Primer (5′→3′)	Tm (°C)	Amplicon (bp)
Human *KLF7*	AGACATGCCTTGAATTGGAACG	GGGGTCTAAGCGACGGAAG	60	137
Human *INS*	GCAGCCTTTGTGAACCAACAC	CCCCGCACACTAGGTAGAGA	60	67
Human *GLUT2*	GCCTGGTTCCTATGTATATCGGT	GCCACAGATCATAATTGCCCAAG	60	147
Human *GAPDH*	GGAGCGAGATCCCTCCAAAAT	GGCTGTTGTCATACTTCTCATGG	60	197
Mouse *Klf7*	TGGACAGAGCGACAGTGAGCAAGGA	CTGTGTGTTTCCTGTAGTGCCTTGT	60	236
Mouse Ins1	CACTTCCTACCCCTGCTGG	ACCACAAAGATGCTGTTTGACA	60	159
Mouse *Ins2*	GCTTCTTCTACACACCCATGTC	AGCACTGATCTACAATGCCAC	59	147
Mouse *Glut2*	TCATTGCTGGACGAAGTGTATC	ACATTGGAACCAGTCCTGAAA	59	63
Mouse *Gapdh*	AGGTCGGTGTGAACGGATTTG	TGTAGACCATGTAGTTGAGGTCA	60	123

### 4.4. Western Blotting

Isolated islets and INS1 cells were lysed in RIPA lysis buffer (Cat# P0013B, Beyotime, Shanghai, China) supplemented with 1× protease inhibitor cocktail and incubated on ice for 30 min, with vortexing every 5 min. Lysates were centrifuged at 12,000× *g* for 15 min at 4 °C, and the supernatants were collected. Protein concentrations were determined using a BCA protein assay kit (Cat# PC0020, Solarbio, Shanghai, China). Equal amounts of protein (25 μg) were mixed with 5× SDS-PAGE loading buffer, denatured at 95 °C for 5 min, separated by 10–12% SDS-PAGE (80 V for the stacking gel and 120 V for the resolving gel) and transferred onto PVDF membranes (Millipore, Burlington, MA, USA) by wet transfer at 300 mA for 60 min. The membranes were blocked with 5% non-fat milk in Tris-buffered saline containing 0.1% Tween 20 (TBST) for 1 h at room temperature (RT) and incubated overnight at 4 °C with primary antibodies against KLF7 (1:1000, Cat# A8879, ABclonal, Wuhan, China), GLUT2 (1:1000, Cat# A12307, ABclonal) or GAPDH (1:10,000, Cat# A19056, ABclonal). After three washes with TBST for 10 min each, the membranes were incubated with HRP-conjugated goat anti-rabbit IgG (H + L) (1:10,000, Cat# AS014, ABclonal) for 1 h at RT. After three further washes with TBST for 10 min each, protein bands were visualised with an ECL substrate and images were captured on a Tanon 5200 imaging system (Tanon, Shanghai, China). Band intensities were quantified using ImageJ (version 2), and target protein levels were normalised to GAPDH.

### 4.5. Measurements of Insulin Secretion

The cells were washed three times with Krebs–Ringer buffer (KRB) with 0.2% BSA 48 h after being transduced. They were then incubated for 2 h in the same buffer. Next, the cells were placed in a KRB solution containing 0.2% BSA and either 0, 2.8 or 16.7 mM glucose. The cells were then left to incubate for 1 h at 37 °C. The incubation media were then collected to measure the amount of insulin secreted by the cells. Finally, Insulin in the incubation medium was quantified by ELISA (Elabscience, Wuhan, Hubei, China) according to the manufacturer’s instructions. The kits used were commercially available. They were used according to the manufacturer’s specifications.

### 4.6. Mouse Metabolic Phenotyping

Glucose tolerance test (GTT) and insulin tolerance test (ITT) were conducted. For GTT, glucose was injected into the mice at a dose of 1.5 g/kg body weight via the intraperitoneal route after they had been fasted for 16 h. For ITT, insulin was injected into the mice at a dose of 0.75 U/kg body weight via the intraperitoneal route after they had been fasted for 4 h. For blood glucose measurements, blood was taken from the tail vein. Blood glucose was analysed using an Accu-Chek glucometer (Roche, Indianapolis, IN, USA). To measure plasma insulin, blood was taken from the retro-orbital sinus, and plasma was separated by centrifugation. Plasma insulin concentration was quantified by ELISA (Elabscience, Wuhan, Hubei, China) according to the manufacturer’s instructions.

### 4.7. Luciferase Reporter Assay

A concentration of 1000 ng per well of pGL4 vector was used for transfecting 293T cells with either the PIRM-control or the PIRM-Klf7 vector. Lysis of the cells was achieved with 100 μL cold 1× PLB lysis buffer (Promega, Madison, WI, USA), followed by shaking for 15 min at room temperature. The cell lysis was then performed using a centrifuge at 12,000 × *g* for 2 min, after which 20 μL of the resulting supernatant was used for a luciferase activity assay (Promega) according to the manufacturer’s instructions.

### 4.8. 2-NBDG Glucose Uptake Assay

Glucose uptake in INS1 cells was assessed using the fluorescent glucose analogue 2-NBDG (2-[N-(7-nitrobenz-2-oxa-1,3-diazol-4-yl)amino]-2-deoxy-D-glucose; Amgicam, Shanghai, China; ajci4820). INS1 cells seeded on glass-bottom dishes were transduced with Klf7 shRNA or control shRNA lentivirus as described in Section 4.3. Seventy-two hours after transduction, the cells were washed three times with containing 0.2% BSA and starved for 1 h at 37 °C in low-glucose KRB (2.8 mM glucose) to deplete intracellular glucose pools. The cells were then incubated with 100 μM 2-NBDG in KRB containing 0.2% BSA for 30 min at 37 °C in the dark. Uptake was terminated by three washes with ice-cold PBS, and the cells were fixed in 4% paraformaldehyde for 15 min at room temperature. After washing with PBS, the cells were imaged immediately on a fluorescence microscope (Olympus, Tokyo, Japan), with identical exposure time, gain and other acquisition settings for all groups within an experiment. At least 10 random, non-overlapping fields were captured per group, and the mean fluorescence intensity per cell was measured in ImageJ, with no fewer than 200 cells analysed per group. Background fluorescence was determined from cell-free regions and subtracted from all values. Fluorescence values are expressed relative to control shRNA cells within the same experiment. Three independent experiments were performed, and data were analysed by an unpaired two-tailed Student’s *t*-test.

### 4.9. Statistical Analysis

Statistical analyses were performed using GraphPad Prism (version 8). Data are presented as means ± s.e.m. unless otherwise indicated. Comparisons between two groups were performed using unpaired two-tailed Student’s *t*-test. Glucose tolerance test and insulin tolerance test time-course data were analysed by two-way repeated-measures ANOVA with Sidak’s post hoc test for multiple comparisons. Area under the curve (AUC) values were compared by unpaired two-tailed Student’s *t-*test. RNA-seq differential expression analysis was performed using DESeq2 with Benjamini–Hochberg correction for multiple testing. For the discovery-oriented analyses (Figure 4), DEGs were defined by |log_2_FC| > 1.0 combined with a nominal *p* < 0.05 to prioritise candidate pathways; for confirmatory analyses, such as the Klf-compensation assessment (Figure S3), the Benjamini–Hochberg-adjusted *p* (*padj* < 0.05) was used. *p* value < 0.05 was considered statistically significant. All n values refer to biologically independent samples or animals. For the human laser-capture microdissected β-cell dataset (GSE20966), the donor characteristics available are those reported in the original study; fasting glucose, HbA1c and HOMA-derived indices were not reported, so donor-level information was insufficient for a full multivariable adjustment and the comparison shown in Figure 1A is unadjusted (Table S1). Before pooling of the four mouse datasets (Figure 1B), *Klf7* expression was normalised within each dataset to the mean of its own WT or lean control (WT = 1.0), so that between-dataset differences in platform, library size and baseline expression do not contribute to the pooled estimate; per-dataset effect sizes and normalisation details are given in Table S2. Because the underlying models, platforms and metabolic stages differ, this pooled analysis is exploratory and is presented as a complement to the dataset-specific findings.

## Figures and Tables

**Figure 1:**
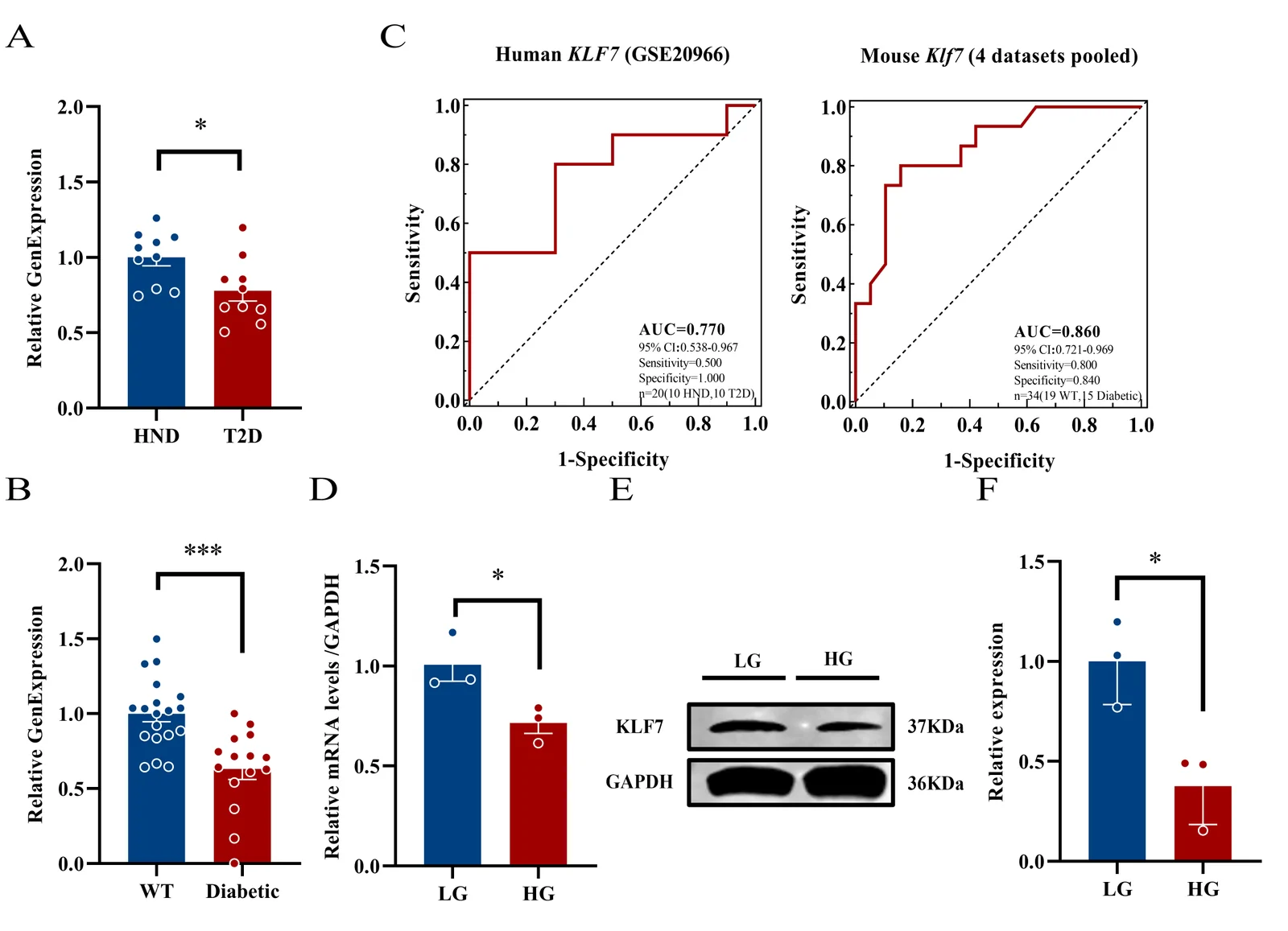
KLF7 expression is downregulated in diabetic β-cells and suppressed by high glucose. (**A**) *KLF7* mRNA levels in laser-capture microdissected (LCM) pancreatic β-cells from human non-diabetic (HND, n = 10) and type 2 diabetic (T2D, n = 10) individuals (GSE20966). Data are means ± s.e.m.; * *p* < 0.05, unpaired Student’s *t*-test. (**B**) Pooled analysis of *Klf7* expression across four independent mouse diabetic transcriptomic datasets (GSE224797 ob/ob β-cells, n = 10 control/5 diabetic; GSE330587 islets of control group from Pax4-R192H knock-in mice fed a high-fat diet, n = 3 control/3 diabetic; GSE294866 high-fat diet β-cells, n = 3 control/4 diabetic; GSE107489 db/db islets, n = 3 control/3 diabetic). Within each dataset, expression values were normalised to the respective WT or lean control (WT = 1.0) before pooling; per-dataset effect sizes and normalisation details are given in Table S2. Values in diabetic samples were 0.63 ± 0.07 vs. 1.00 ± 0.05 in controls (*** *p* < 0.001, n = 19 control/15 diabetic, unpaired Student’s *t*-test). Because the underlying models, platforms and metabolic stages differ, this pooled analysis is exploratory. (**C**) ROC curves for KLF7 expression in human LCM β-cells (left) and mouse islets (right). Human: AUC = 0.770 (10/group, 95% CI: 0.538–0.967); mouse (pooled): AUC = 0.860 (n = 19 control/15 diabetic, 95% CI: 0.721–0.969). Cut-off was determined by the Youden index. (**D**) *Klf7* mRNA levels in isolated mouse islets were treated with low glucose (LG, 5.5 mM) or high glucose (HG, 25 mM) for 48 h, measured by RT-PCR and normalised to Gapdh (n = 3 independent experiments). Data are means ± s.e.m.; * *p* < 0.05. (**E**,**F**) KLF7 protein levels in isolated mouse were islets treated with LG or HG for 48 h, assessed by Western blot and normalised to GAPDH (n = 3 independent experiments). Data are means ± s.e.m.; * *p* < 0.05.

**Figure 2:**
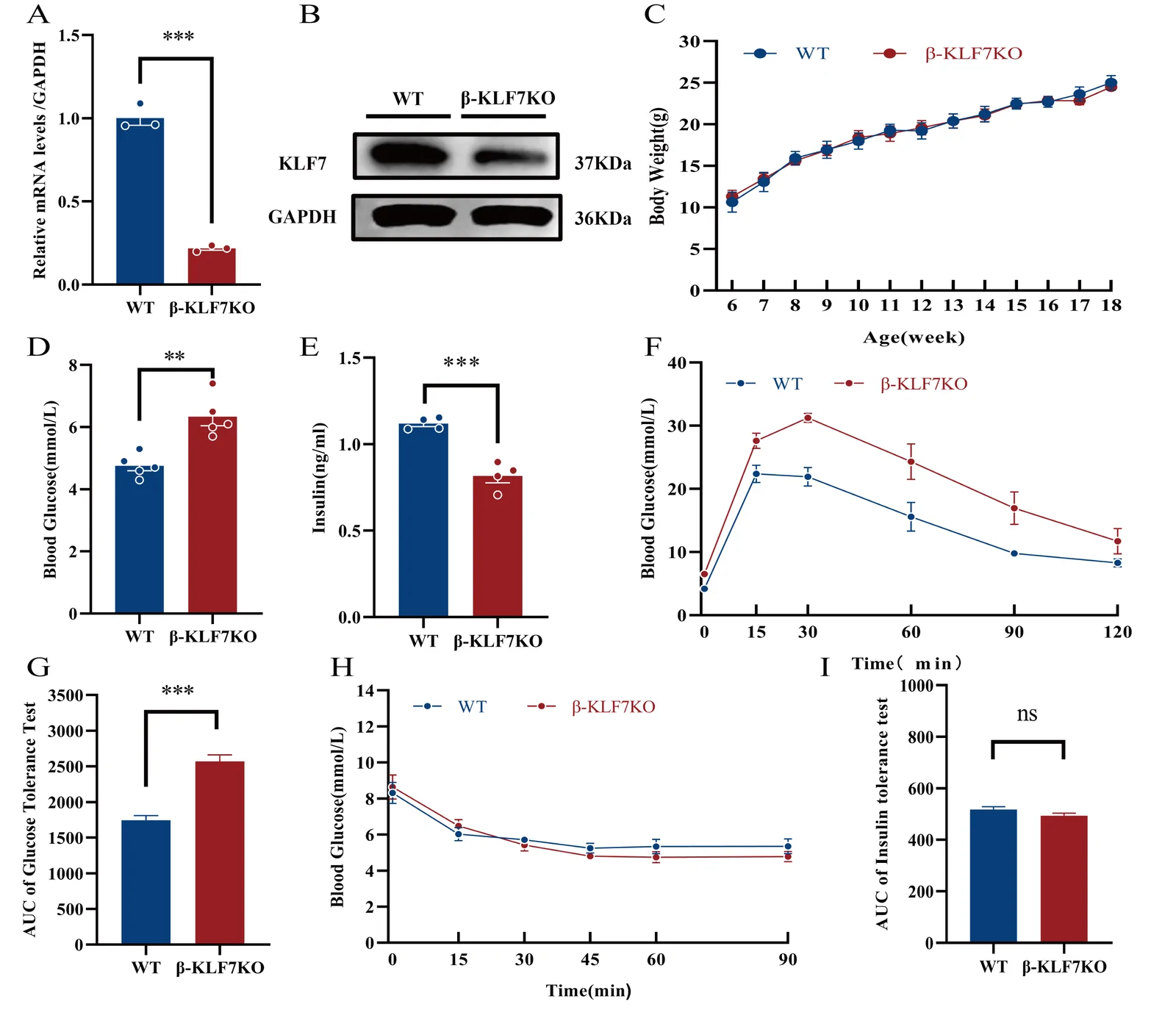
β-cell-specific deletion of *Klf7* impairs glucose tolerance and insulin secretion. (**A**) *Klf7* mRNA levels in pancreatic islets from *Klf7*^fl/fl^ (WT, n = 3) and β-KLF7KO (n = 3) mice, measured by RT-PCR and normalised to *Gapdh*. Data are means ± s.e.m.; *p* < 0.001, unpaired Student’s *t*-test. (**B**) Representative immunoblot of KLF7 protein in islets from WT and β-KLF7KO mice (n = 3). GAPDH served as loading control. (**C**) Body weight of WT and β-KLF7KO mice measured weekly from 6 to 18 weeks of age. n ≥ 5. Data are means ± s.e.m. (**D**) Fasting blood glucose levels in 12-week-old WT and β-KLF7KO mice. n ≥ 5. Data are means ± s.e.m.; *p* < 0.01, unpaired Student’s *t*-test. (**E**) Fasting plasma insulin levels in WT and β-KLF7KO mice. n = 4. Data are means ± s.e.m.; *p* < 0.01, unpaired Student’s *t*-test. (**F**,**G**) Intraperitoneal glucose tolerance test (GTT, 1.5 g/kg body weight) (**F**) and area under the curve (AUC) (**G**). WT and β-KLF7KO mice. n ≥ 4. GTT curves: two-way repeated-measures ANOVA with Sidak’s post hoc test; AUC: unpaired Student’s *t-*test. (**H**,**I**) Insulin tolerance test (ITT, 0.75 U/kg) (**H**) and AUC (**I**). WT (n = 7) and β-KLF7KO (n = 5) mice. n ≥ 5. No detectable difference in insulin sensitivity (genotype × time interaction, two-way repeated-measures ANOVA, *p* = 0.42; AUC, unpaired Student’s *t*-test, *p* = 0.13). All bar graphs show individual data points with mean ± s.e.m. ** *p* < 0.01, *** *p* < 0.001; ns, not significant.

**Figure 3:**
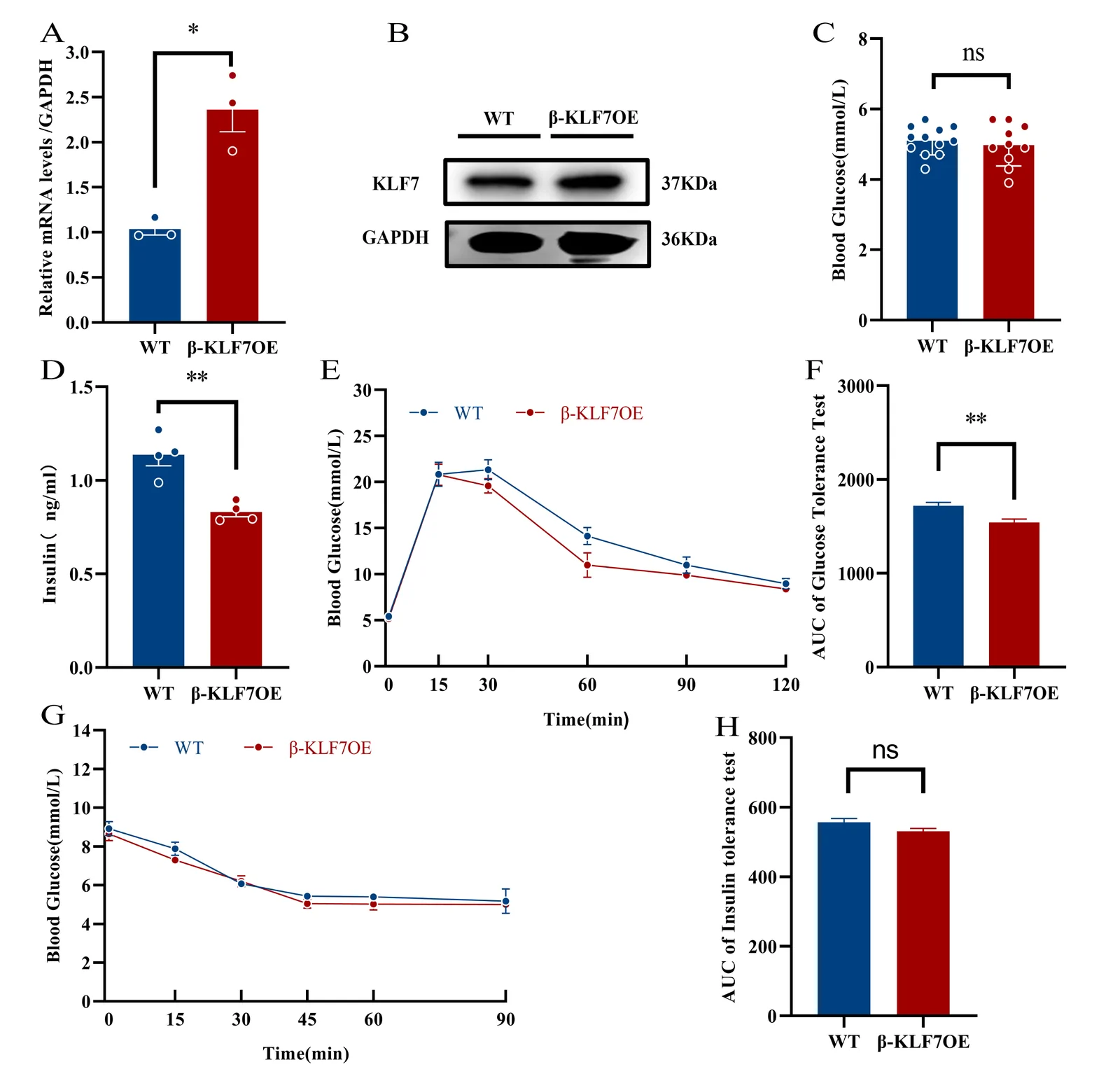
β-cell-specific *Klf7* overexpression alters glucose homeostasis. (**A**) *Klf7* mRNA levels in pancreatic islets from WT and β-KLF7OE mice, measured by RT-PCR and normalised to *Gapdh*. n = 3. Data are means ± s.e.m.; *p* < 0.05, unpaired Student’s *t*-test. (**B**) Representative immunoblot of KLF7 protein in islets from WT and β-KLF7OE mice. (**C**) Fasting blood glucose levels in WT and β-KLF7OE mice. n ≥ 10. Data are means ± s.e.m.; ns, not significant, unpaired Student’s *t*-test. (**D**) Fasting plasma insulin levels in WT and β-KLF7OE mice. n = 4. Data are means ± s.e.m.; *p* < 0.01, unpaired Student’s *t*-test. (**E**,**F**) Intraperitoneal glucose tolerance test (GTT, 1.5 g/kg body weight) (**E**) and area under the curve (AUC) (**F**). WT and β-KLF7OE mice. n ≥ 6. GTT curves: two-way repeated-measures ANOVA with Sidak’s post hoc test; AUC: *p* < 0.01, unpaired Student’s *t*-test. (**G**,**H**) ITT (0.75 U/kg) (**G**) and AUC (**H**). WT and β-KLF7OE mice. n = 4. ITT curves: genotype × time interaction, two-way repeated-measures ANOVA; AUC: no significant difference, unpaired Student’s *t*-test. All bar graphs show individual data points with mean ± s.e.m. * *p* < 0.05, ** *p* < 0.01; ns, not significant.

**Figure 4:**
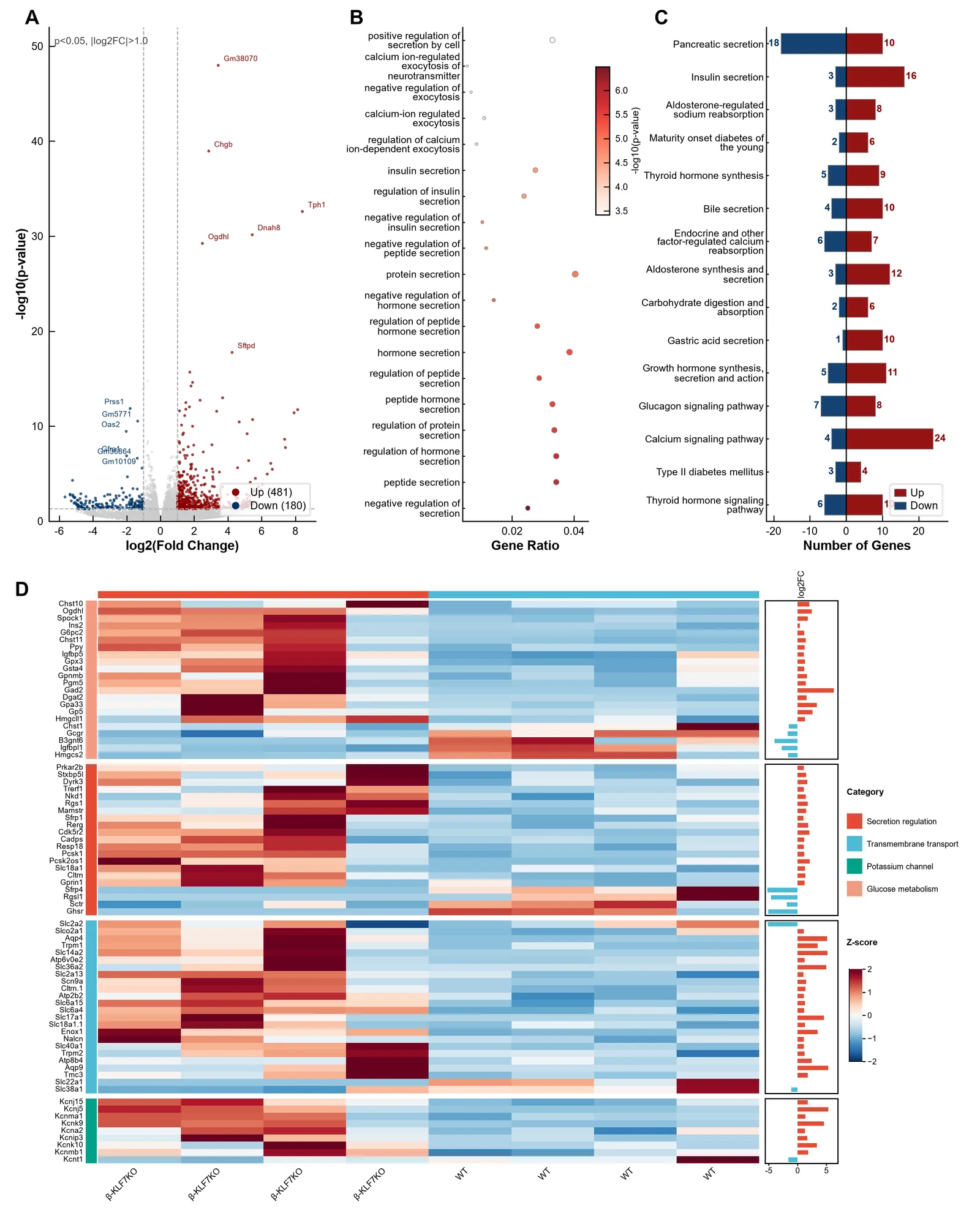
Transcriptomic analysis of islets from β-KLF7KO mice reveals pervasive dysregulation of insulin secretion and glucose metabolism pathways. (**A**) Volcano plot showing differentially expressed genes (DEGs) in β-KLF7KO vs. WT islets. Thresholds: |log_2_FC| > 1.0, *p* < 0.05, an exploratory, discovery-oriented criterion used to prioritise candidate pathways. Red, upregulated genes (n = 481); blue, downregulated genes (n = 180). n = 4. (**B**) GO enrichment analysis of DEGs. Bubble size represents gene count; colour represents −log_10_ (*p* value). Top enriched biological processes include insulin secretion, glucose metabolism, and transmembrane transport. (**C**) KEGG pathway enrichment analysis showing the distribution of upregulated and downregulated DEGs across significantly enriched pathways. The insulin secretion pathway was prominently affected. (**D**) Integrated heatmap of DEGs enriched in four functional categories that were jointly dysregulated in β-KLF7KO islets: secretion regulation, transmembrane transport, glucose metabolism, and potassium channel activity. Genes are grouped by category (colour bars on the left) and each row represents an individual gene; each column represents an individual mouse (β-KLF7KO1–4, n = 4; WT1–4, n = 4), with genotype indicated by the annotation bar above the heatmap. Cell colours represent row-scaled z-scores of normalised expression (–2 to +2); horizontal bars on the right show the log_2_FC (β-KLF7KO vs. WT) for each gene. All RNA-seq data are from n = 4 biologically independent samples per group; DEGs were identified using DESeq2 with an exploratory threshold (|log_2_FC| > 1.0, *p* < 0.05).

**Figure 5:**
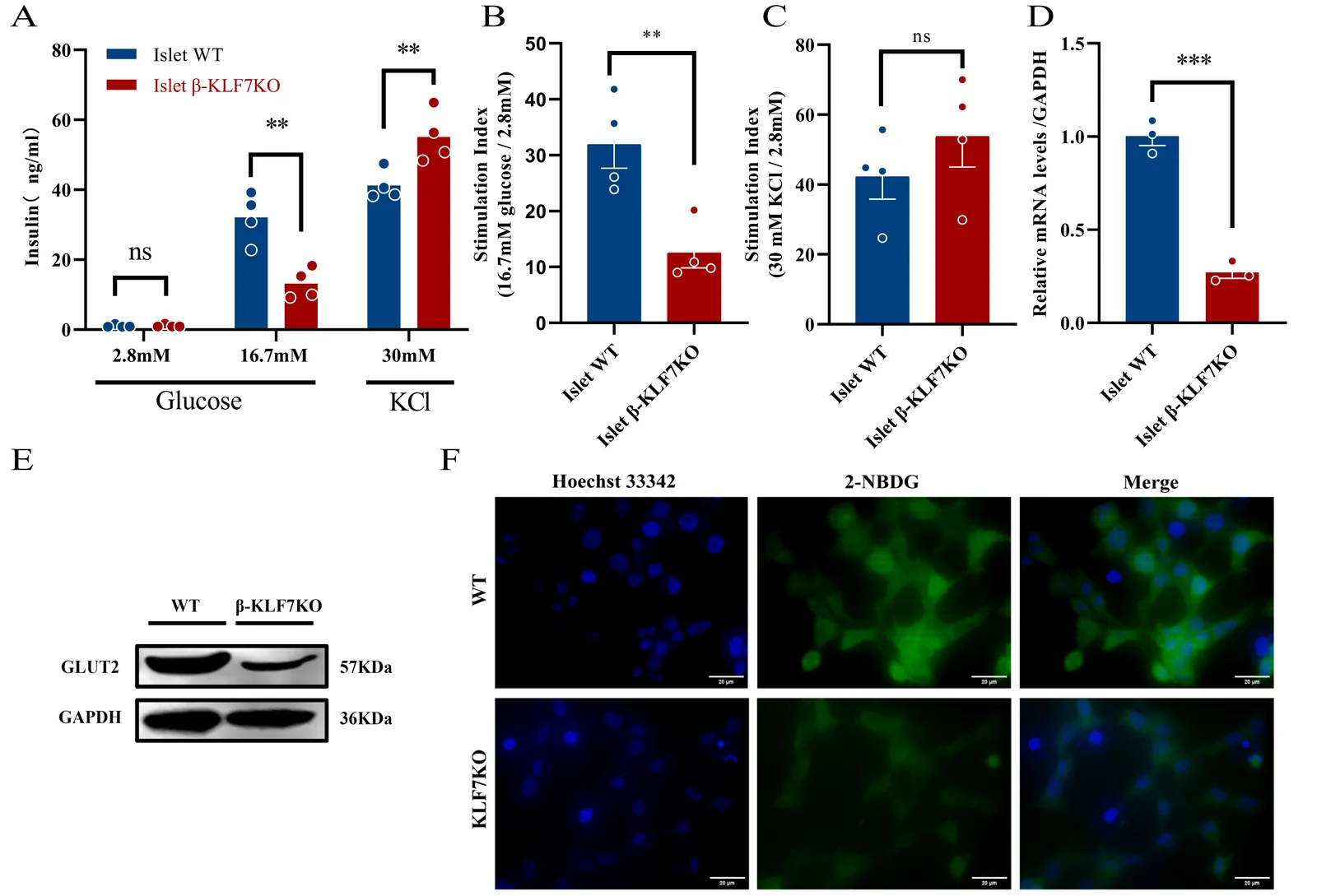
Klf7 deficiency impairs glucose uptake in β-cells. (**A**) GSIS from isolated WT and β-KLF7KO islets. Islets were incubated with 2.8 mM glucose, 16.7 mM glucose, and 30 mM KCl. β-KLF7KO islets showed significantly reduced insulin secretion at 16.7 mM glucose, whereas KCl-stimulated secretion was preserved. Data are means ± s.e.m.; n = 4 independent experiments per group. ** *p* < 0.01, two-way repeated-measures ANOVA with Sidak’s post hoc test. (**B**) Glucose stimulation index (SI = insulin at 16.7 mM glucose/insulin at 2.8 mM glucose). n = 4, ** *p* < 0.01, unpaired Student’s *t*-test. (**C**) KCl stimulation index (SI = insulin at 30 mM KCl/insulin at 2.8 mM glucose). ns: No significant difference was observed. *p* = 0.33, unpaired Student’s *t*-test. (**D**) *Glut2* mRNA levels in isolated WT and β-KLF7KO islets, measured by RT-PCR and normalised to *Gapdh*. n = 3. Data are means ± s.e.m. *** *p *< 0.001, unpaired Student’s *t*-test. (**E**) Representative immunoblot and densitometric quantification of GLUT2 protein in WT and β-KLF7KO islets. GAPDH served as loading control. n = 3. (**F**) 2-NBDG glucose uptake assay in INS1 cells transduced with control or *Klf7* shRNA. Scale bar, 50 μm. n = 3. All bar graphs show individual data points with mean ± s.e.m.

**Figure 6:**
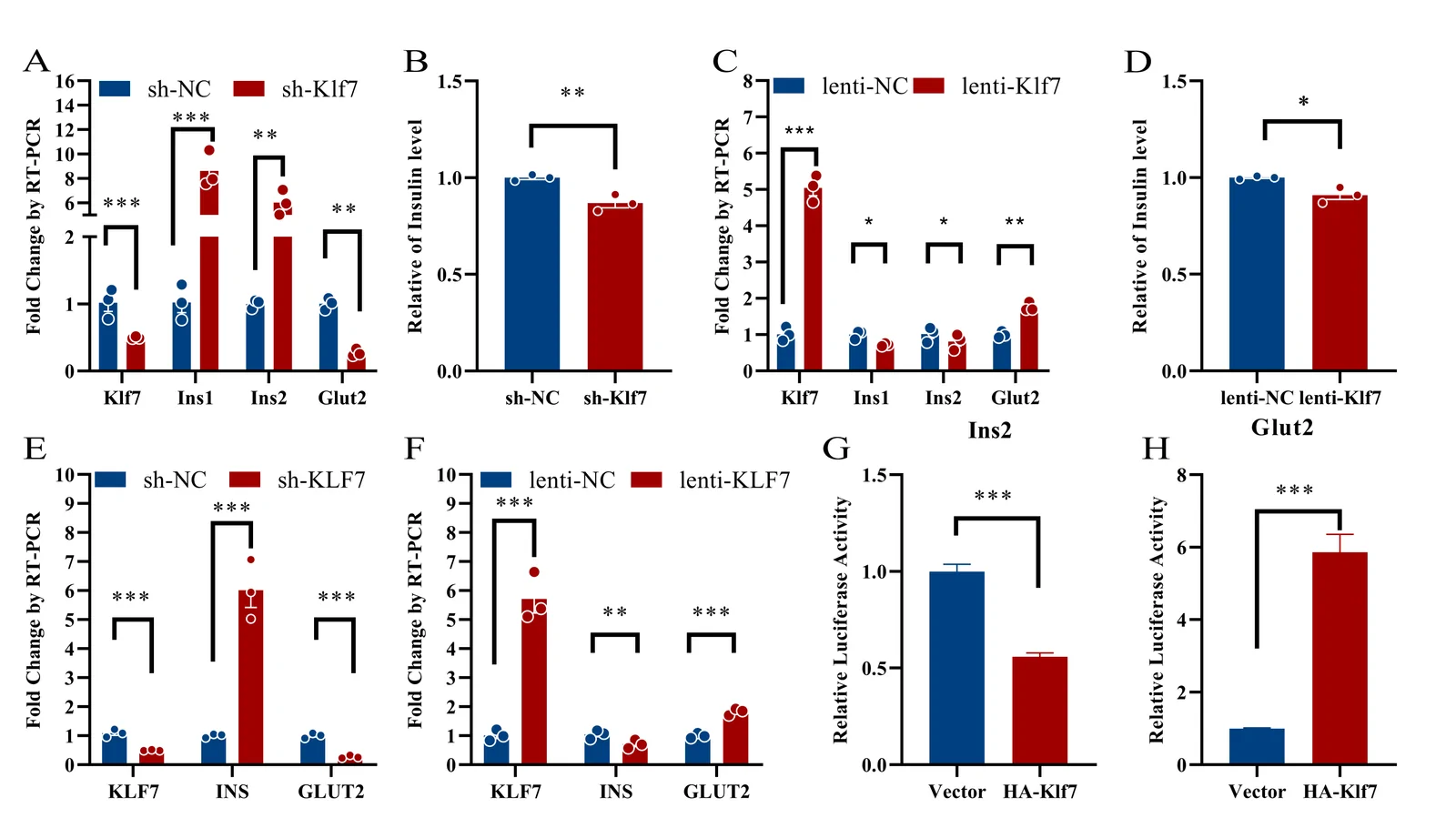
*Klf7* directly regulate*s Glut2* and *Ins2* expression in β-cells. (**A**) mRNA levels of *Klf7*, *Ins1, Ins2* and *Glut2* in INS1 cells transduced with control (sh-NC) or *Klf7* shRNA (sh-*Klf7*), measured by RT-PCR and normalised to *Gapdh*. *Klf7* knockdown derepressed *Ins1* and *Ins2* while suppressing *Glut2*. n = 3. Data are means ± s.e.m.; independent experiments; ** *p* < 0.01, *** *p* < 0.001, unpaired Student’s *t*-test. (**B**) Total insulin secretion from INS1 cells after *Klf7* knockdown. Despite increased *Ins1* and *Ins2* mRNA levels, secreted insulin was modestly reduced by ~13%. n = 3. Data are means ± s.e.m.; independent experiments; ** *p* < 0.01, unpaired Student’s *t*-test. (**C**) mRNA levels of *Klf7*, *Ins1*, *Ins2* and *Glut2* in INS1 cells transduced with control (lenti-NC) or *Klf7* overexpression (lenti-*Klf7*) lentivirus. *Klf7* overexpression suppressed *Ins1* and *Ins2* while upregulating *Glut2*. n = 3. Data are means ± s.e.m.; * *p* < 0.05, ** *p* < 0.01, *** *p* < 0.001, unpaired Student’s *t*-test. (**D**) Total insulin secretion from INS1 cells after *Klf7* overexpression. Insulin secretion was reduced, consistent with decreased Ins mRNA levels. n = 3. Data are means ± s.e.m.; * *p* < 0.05, unpaired Student’s *t*-test. (**E**) mRNA levels of *KLF7*, *INS* and *GLUT2* in the 1.1B4 human pancreatic islet fusion cell line transduced with sh-NC or sh-*KLF7*. *KLF7* knockdown upregulated *INS* and downregulated *GLUT2*, recapitulating the regulatory pattern observed in mouse INS1 cells. n = 3. Data are means ± s.e.m.; *** *p* < 0.001, unpaired Student’s *t*-test. (**F**) mRNA levels of *KLF7*, *INS* and *GLUT2* in 1.1B4 cells transduced with lenti-NC or lenti-*KLF7*. *KLF7* overexpression suppressed *INS* and upregulated *GLUT2*. n = 3. Data are means ± s.e.m.; ** *p* < 0.01,*** *p* < 0.001, unpaired Student’s *t*-test. (**G**) Luciferase reporter assay showing the effect of *KLF7* on *Ins2* promoter activity in 293T cells. n = 3. Data are means ± s.e.m.; *** *p* < 0.001, unpaired Student’s *t*-test vs. vector control. (**H**) Luciferase reporter assay showing the effect of *KLF7* on *Glut2* promoter activity. Data are means ± s.e.m.; n = 3 independent experiments; *** *p* < 0.001, unpaired Student’s *t*-test vs. vector control. All bar graphs show individual data points with mean ± s.e.m.

**Figure 7:**
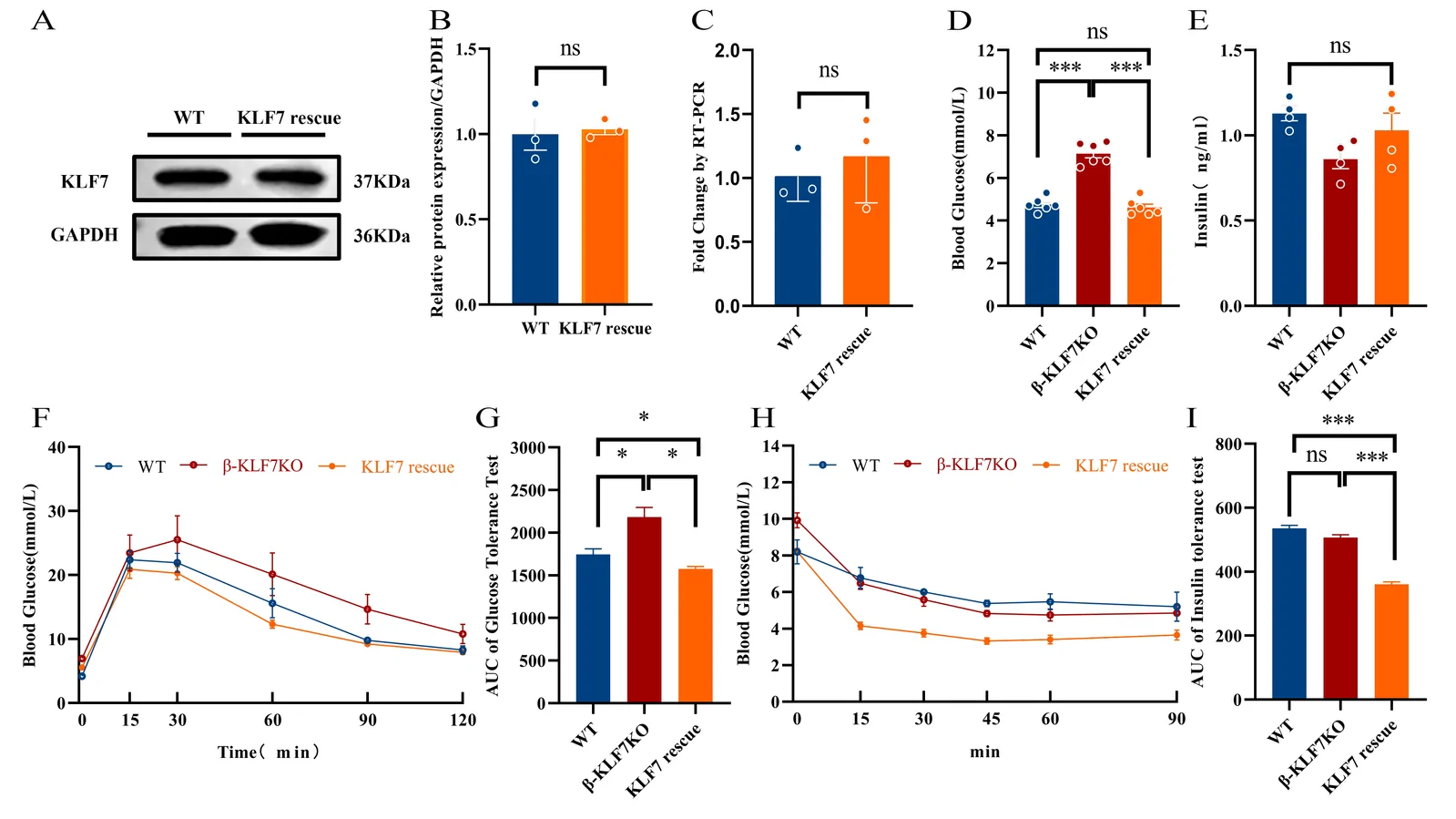
Restoration of *Klf7* in β-KLF7KO mice improves glucose homeostasis. (**A**) Representative immunoblot of KLF7 in WT and *Klf7* rescue islets. GAPDH served as loading control. (**B**) Densitometric quantification of KLF7 protein levels. KLF7 expression was restored to near-WT levels in rescue islets. n = 3. Data are means ± s.e.m. (**C**) *Ins2* mRNA levels in islets from WT and *Klf7* rescue mice, measured by RT-PCR and normalised to *Gapdh*. n = 3. Data are means ± s.e.m. (**D**) Fasting blood glucose levels in WT and *Klf7* rescue mice. Rescue significantly reduced hyperglycaemia. n = 6, *** *p* < 0.001, unpaired Student’s *t*-test. Data are means ± s.e.m. (**E**) Fasting plasma insulin levels in WT and *Klf7* rescue mice. n = 4. Data are means ± s.e.m. (**F**,**G**) Intraperitoneal GTT (1.5 g/kg) (**F**) and AUC (**G**). WT, β-KLF7KO, and *Klf7* rescue mice. n ≥ 4, GTT curves: two-way repeated-measures ANOVA with Sidak’s post hoc test; AUC: * *p* < 0.05, unpaired Student’s *t*-test. (**H**,**I**) ITT (0.75 U/kg) (**H**) and AUC (**I**). WT, β-KLF7KO and *Klf7* rescue mice. n ≥ 4, ITT curves: genotype × time interaction, two-way repeated-measures ANOVA; AUC: *** *p* < 0.001, unpaired Student’s *t*-test. All bar graphs show individual data points with mean ± s.e.m.

**Figure 8:**
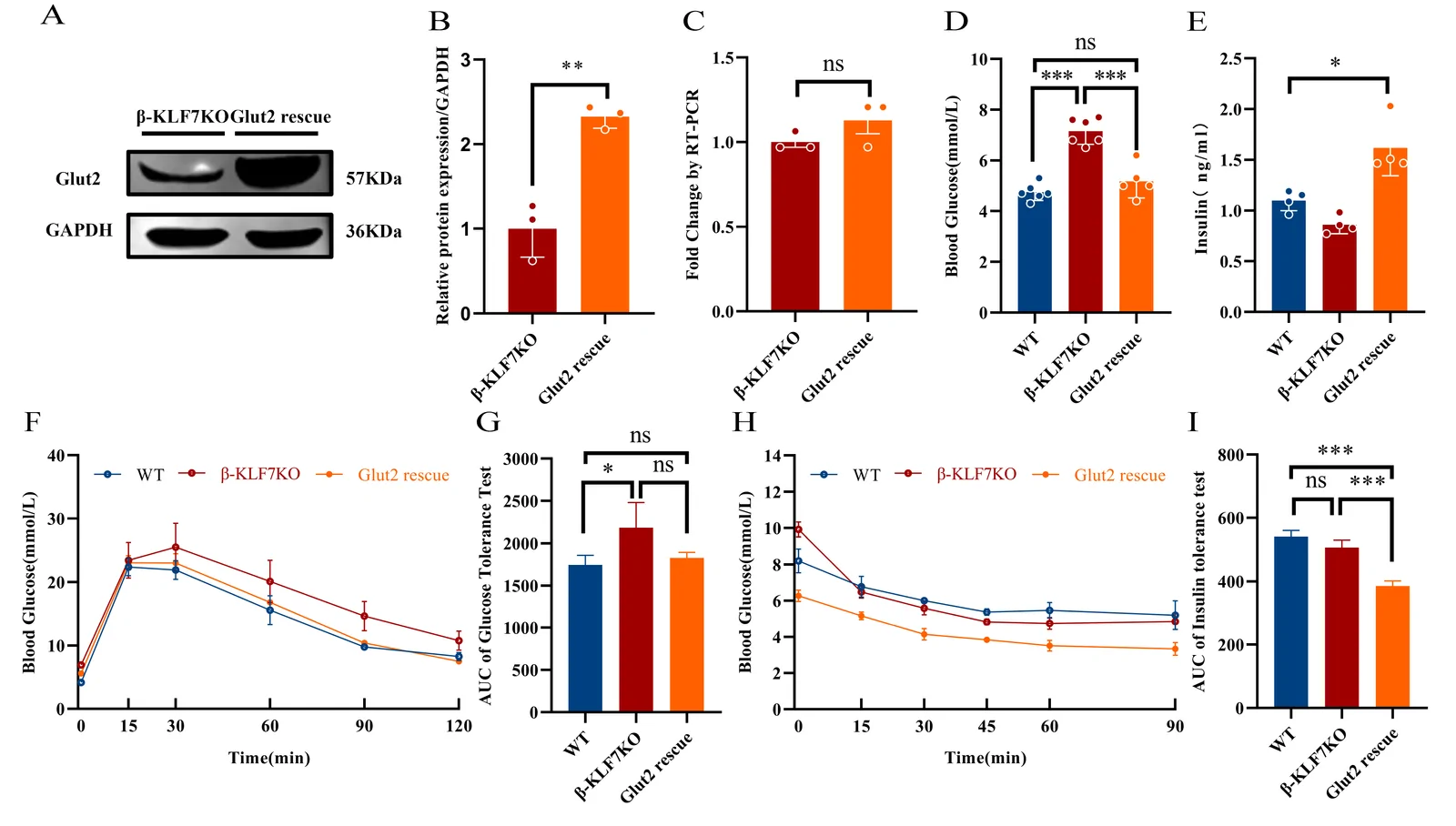
Re-expression of *Glut2* in β-*KLF7*KO mice rescues glucose homeostasis. (**A**) Representative immunoblot of GLUT2 protein in islets from β-KLF7KO and *Glut2* rescue mice. GAPDH served as loading control. (**B**) Densitometric quantification of GLUT2 protein in β-KLF7KO and *Glut2* rescue islets. n = 3. ** *p* < 0.01, Data are means ± s.e.m. (**C**) *Ins2* mRNA levels in islets from β-KLF7KO and *Glut2* rescue mice. *Ins2* expression remained unchanged, confirming that *Glut2* rescue acts downstream of *Klf7* without altering *Ins2* transcription. n = 3. Data are means ± s.e.m.; ns, not significant, unpaired Student’s *t*-test. (**D**) Fasting blood glucose levels in WT, β-KLF7KO and *Glut2* rescue mice. Rescue normalised hyperglycaemia. *** *p* < 0.001, n ≥ 4, unpaired Student’s *t*-test. Data are means ± s.e.m. (**E**) Fasting plasma insulin levels in WT, β-KLF7KO and *Glut2* rescue mice. Rescue insulin levels exceeded WT, suggesting supranormal secretion driven by enhanced glucose uptake. n ≥ 4, * *p* < 0.05, ns, not significant. Data are means ± s.e.m. (**F**,**G**) Intraperitoneal GTT (1.5 g/kg) (**F**) and AUC (**G**). WT, β-KLF7KO and *Glut2* rescue mice. n ≥ 4, GTT curves: two-way repeated-measures ANOVA with Sidak’s post hoc test; AUC: * *p* < 0.05, unpaired Student’s *t*-test. (**H**,**I**) ITT (0.75 U/kg) (**H**) and AUC (**I**). WT, β-KLF7KO and *Glut2* rescue mice. n ≥ 4, ITT curves: genotype × time interaction, two-way repeated-measures ANOVA; AUC: *** *p* < 0.001, unpaired Student’s *t*-test. All bar graphs show individual data points with mean ± s.e.m.

## Data Availability

All relevant data supporting the key findings of this study are available within the article and its Supplementary Information files or from the corresponding author upon reasonable request.

## Supplementary Materials

The following supporting information can be downloaded at: https://www.mdpi.com/article/10.3390/ijms27198553/s1.
