# Supplementary material for: The Klf7/Glut2/Ins2 Axis Regulates Glucose Homeostasis
Source: Int J Mol Sci. 2026 Sep 24;27(19):8553. doi: 10.3390/ijms27198553 (PMC13664755; doi:10.3390/ijms27198553)
Supplement: Supplementary file 1 [file ijms-27-08553-s001.zip › ijms-4525888-supplementary.pdf]

Supplementary Figures and Table

The *Klf7*/*Glut2*/*Ins2* Axis Regulates Glucose Homeostasis

Chaofan Yang, Hebing Liu, Cao Wang, Fuxing Zhu, Mingyue Guo, Weiming Tian,*

School of Life Science and Technology, Harbin Institute of Technology, 150080 Harbin, China

Table S1. Clinical characteristics of type 2 diabetic and control subjects.

	Controls(HND)	Type 2 diabetic donors(T2D)
Diagnostic Criteria	-	FPG≥7.0 mmol/L；<br>OGTT≥11.1 mmol/L；<br>HbA1c≥6.5%；<br>RPG≥11.1 mmol/L
Gender（Males/Females）	6/4	7/3
Age(years)	60±5	67±7
BMI(Kg/m²)	30.5±6.5	30.9±6.2(n=9)
Race	White	White
Duration of diabetes	-	5.3±2.3(n=7,data of 3 unavailable)
Anti-diabetic therapy	-	Oral anti-diabetic therapy n=6;insulin treated n=3
Cause of death	8CVD,1TR,1SA	8CVD,1TR
FBG / HbA1c / HOMA	NR	NR
Disease Stage	-	Diagnosed with T2D and receiving hypoglycemic therapy, in the phase of β-cell dysfunction (typical T2D phenotype range, including amyloid deposition).

Diagnostic Criteria :clinically diagnosed T2D; specific thresholds not reported in the source study. We provide international diabetes standards here.

Table S2. Characteristics of the four publicly available mouse islet transcriptome datasets compared in this study.

	GSE107489	GSE224797	GSE330587	GSE294866
Sample	Isolated islets	FACS-sorted β cells	Isolated islets (bulk RNA-seq)	Isolated islets (bulk RNA-seq)
Groups (n)	WT / db+ / db/db (n = 3 each)	lean 10 vs ob/ob 5 (β cells)	Pax4-WT(HFD) vs Pax4-HH(HFD) (n = 3 each)	Chow + vehicle (n = 3) vs HFD + vehicle (n = 4)
Model	Lepr db/db (BKS background)	$Lep^{ob/ob}$	Pax4-R192H knock-in + HFD	60% HFD
Sex(Males/Females)	NR	lean 5 M + 5 F; ob/ob 2 M + 3 F	Male	Male
Age	12 weeks	35–38 weeks	HFD from 12 wk, sampled at 16 wk	from 8 wk
Diet / duration	Inherited (db/db), 12 wk	Inherited (ob/ob), long-standing obesity	D12492 (60% kcal fat) × 4 wk	2 wk HFD then 4 wk treatment[3]
Glycaemic / insulin data	NR	NR	Random blood glucose, GTT (16 h), ITT, GSIS, plasma insulin;	Blood glucose (biweekly), GTT (6 h), serum insulin ELISA;
Disease stage[5]	Overt diabetes, β cell dedifferentiation	Obesity with insulin resistance (transition to decompensation)	Impaired glucose tolerance and reduced insulin secretion	Diet-induced obesity/insulin resistance and β cell senescence

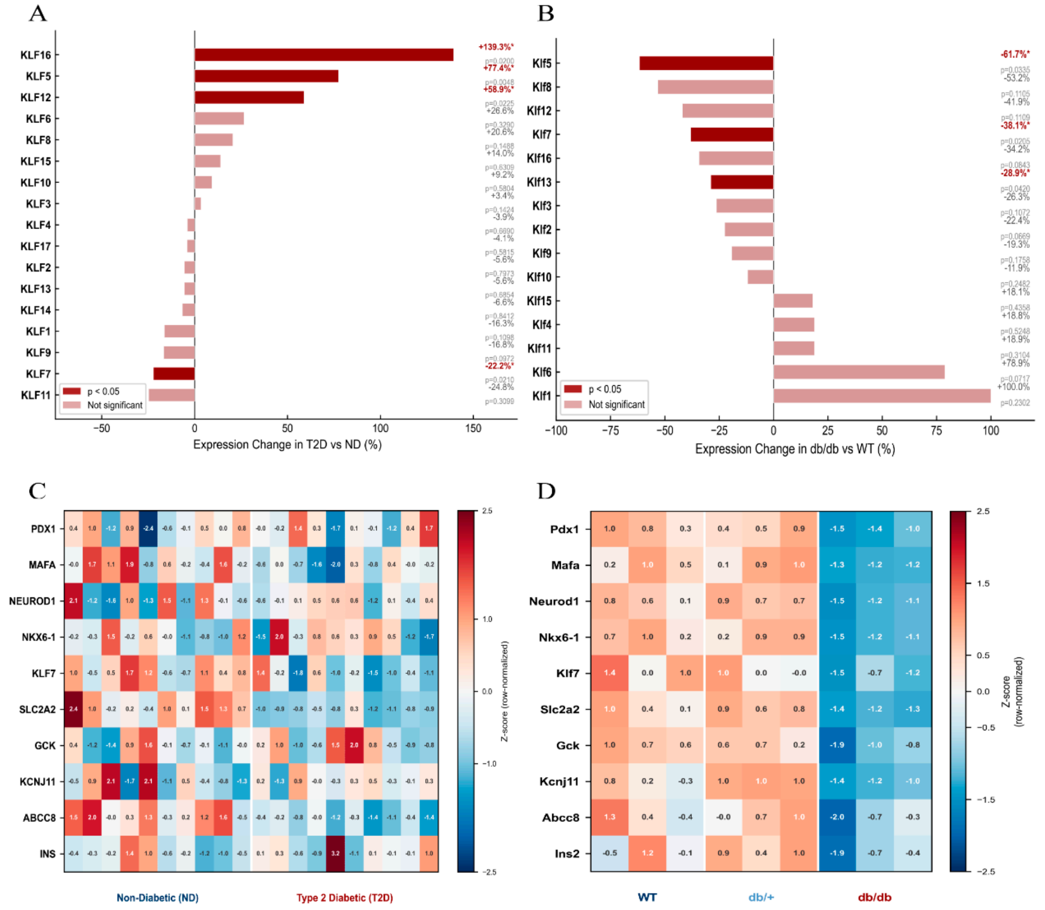

Figure S1. *KLF7* is selectively downregulated among *KLFs* and correlates with β-cell functional genes. (A) Expression of all detectable *KLFs* in human LCM β-cells (GSE20966, n = 10 ND, n = 10 T2D). Red bars indicate $p < 0.05$; grey bars indicate not significant; (B) Expression of *Klfs* in db/db versus WT mouse islets (GSE107489, n = 3 per group). 3 of 15 Klf genes were significantly downregulated; *Klf7* showed the reduction (-38.1%, $p = 0.0143$). Red bars indicate $p < 0.05$; grey bars indicate not significant; (C) Pearson correlation matrix of *KLF7* with nine β-cell functional genes (*SLC2A2*, *ABCC8*, *MAFA*, *PDX1*, *NKX6-1*, *NEUROD1*, *INS*, *KCNJ11*, *GCK*) in human LCM β-cells (n = 20, GSE20966). Values represent Pearson correlation coefficients; (D) Gradient heatmap (row-normalised Z-scores) showing coordinated expression changes of *Klf7* and eight β-cell functional genes across WT, db/+ and db/db mouse islets (GSE107489). Expression values are row-normalised Z-scores.

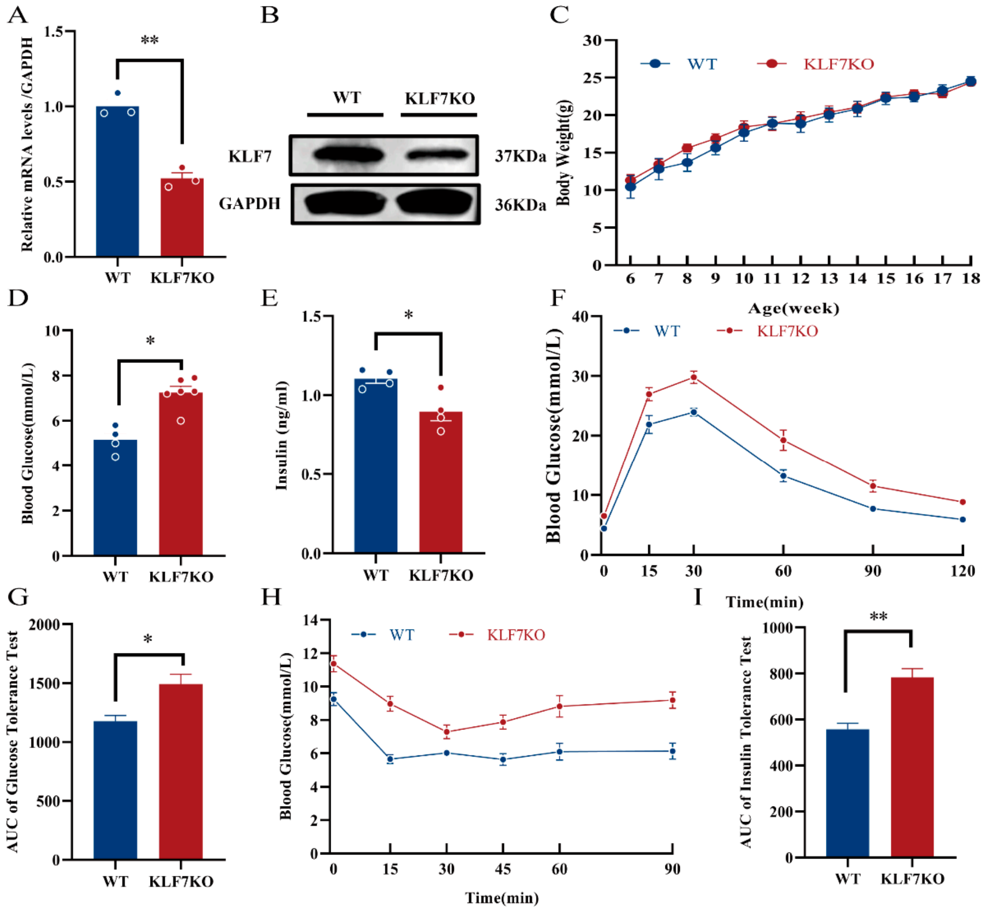

Figure S2. Pancreatic *KLF7* knockout (KLF7KO) impairs glucose homeostasis. (A) *Klf7* mRNA levels in pancreatic islets from *Klf7*$^{fl/fl}$ (WT) and KLF7KO mice, measured by RT-PCR and normalised to *Gapdh*. n = 3. Data are means ± s.e.m.;***p* < 0.01, unpaired Student's t-test; (B) Representative immunoblot of KLF7 protein in pancreas from WT and KLF7KO mice; (C) Body weight of WT (n = 4) and KLF7KO (n = 6) mice from 6 to 18 weeks of age. n ≥ 4. Data are means ± s.e.m; (D) Fasting blood glucose levels in WT and KLF7KO mice. n ≥ 4. Data are means ± s.e.m.; **p* < 0.05, unpaired Student's t-test; (E) Fasting plasma insulin levels in WT (n = 4) and KLF7KO (n = 4) mice. n ≥ 4. Data are means ± s.e.m.; **p* < 0.05, unpaired Student's t-test; (F-G) Intraperitoneal glucose tolerance test (GTT, 1.5 g/kg) (F) and AUC (G). WT and KLF7KO mice. n ≥ 4, GTT curves: two-way repeated-measures ANOVA with Sidak's post-hoc test; AUC: **p* < 0.05, unpaired Student's t-test; (H-I) Insulin tolerance test (ITT, 0.75 U/kg) (H) and AUC (I). WT and KLF7KO mice. n ≥ 4, ITT curves: genotype × time interaction, two-way repeated-measures ANOVA; AUC: ***p* < 0.01, unpaired Student's t-test. All bar graphs show individual data points with mean ± s.e.m.

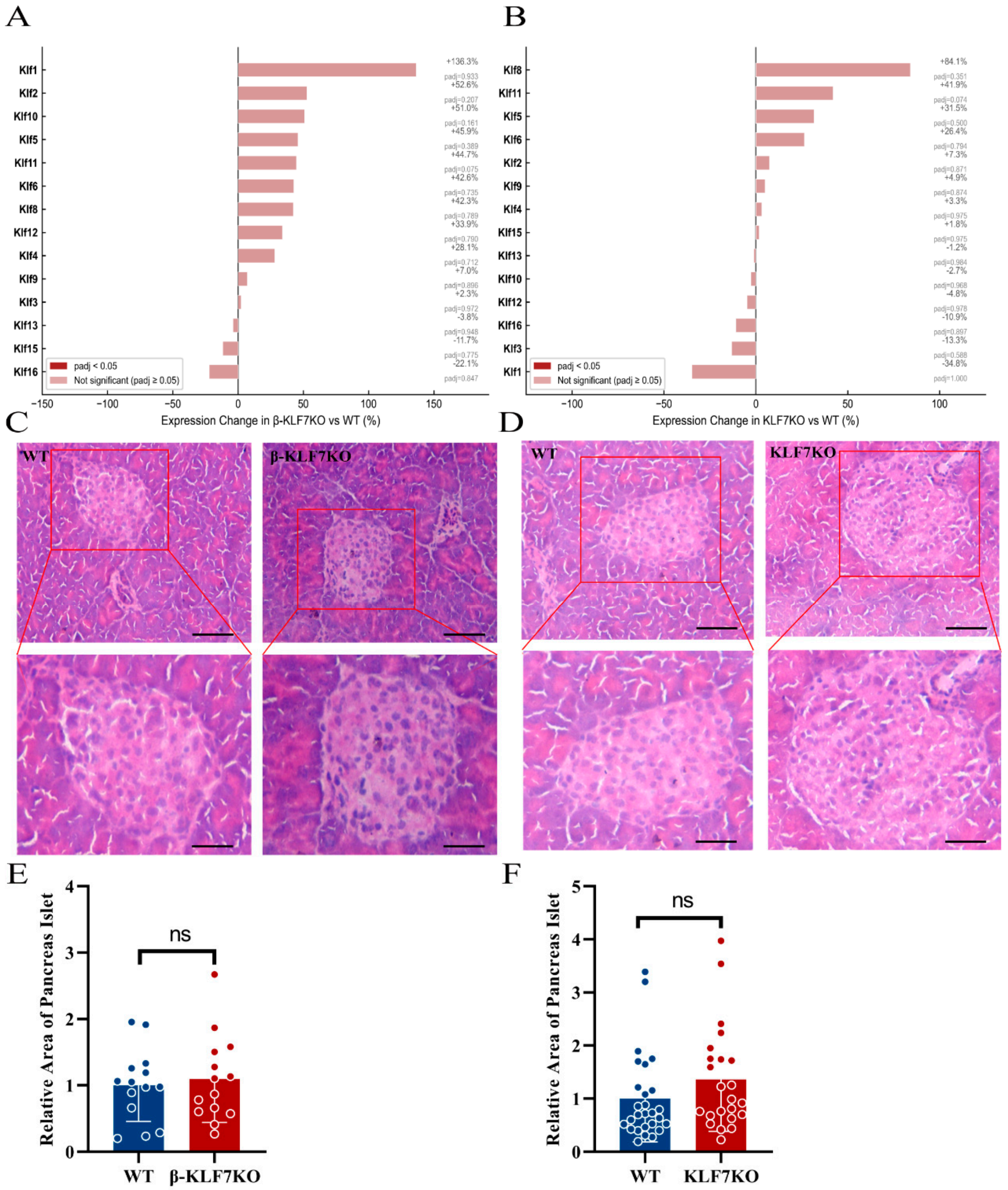

Figure S3. *Klf7* deletion does not induce compensatory changes in other *Klfs* and does not alter islet morphology. (A) Expression of all detectable *Klf* family members in β-KLF7KO versus WT islets, assessed by RNA-seq (n = 4 per group). No gene showed a statistically significant change after multiple-testing correction (DESeq2 with Benjamini–Hochberg adjustment; all *padj* > 0.05). Bars represent percent change relative to WT. Red bars, raw $p < 0.05$ but *padj* > 0.05; grey bars, not significant;(B) Expression of all detectable *Klfs* in KLF7KO versus WT islets (n = 4 per group). No gene showed a statistically significant change after multiple-testing correction (all *padj* > 0.05). Colour coding as in (A); (C, E) Representative H&E staining of pancreatic sections from β-KLF7KO mice (C) and quantification of relative islet area (E). Scale bar 50 μm and 25 μm.

Data are means ± s.e.m.; ns, not significant (unpaired Student's t-test); (D, F) Representative H&E staining of pancreatic sections from KLF7KO mice (D) and quantification of relative islet area (F). Scale bar 50 μm and 25 μm. Data are means ± s.e.m.; ns, not significant (unpaired Student's t-test).

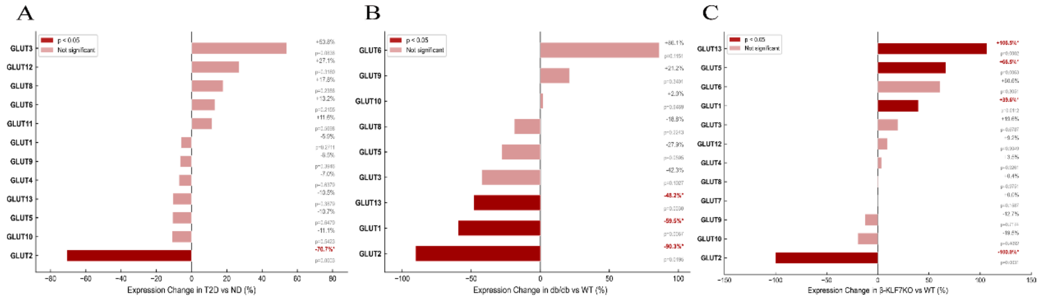

Figure S4. GLUT family expression analysis confirms specific *Glut2* downregulation across datasets. (A) Expression of all detectable *GLUT* family members in LCM human β-cells from T2D versus non-diabetic donors (GSE20966). Only *GLUT2* was significantly downregulated. n = 10. Data are means ± s.e.m.; $p < 0.05$, unpaired Student's t-test; (B) Expression of *Glut* family members in islets from db/db versus WT mice (GSE107489). *Glut2* was the most significantly downregulated *GLUT* member. n = 3. Data are means ± s.e.m.; $p < 0.05$, unpaired Student's t-test;. (C) Expression of *Glut* family members in β-KLF7KO versus WT islets, assessed by RNA-seq. Among all Glut genes, only *Glut2* was significantly downregulated. n = 4. Data are means ± s.e.m.; $p < 0.05$.

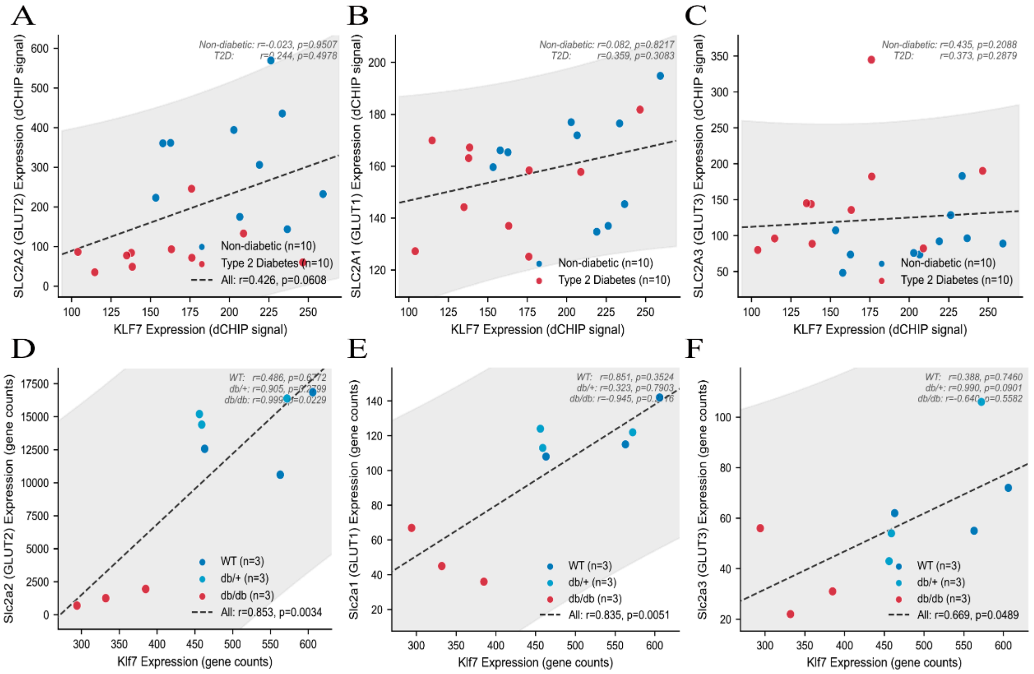

Figure S5. Correlation between *KLF7* and *GLUT* family members in human and mouse islets. (A) Pearson correlation between *KLF7* and *SLC2A2 (GLUT2)* expression in human pancreatic islets from non-diabetic and T2D donors (GSE20966). n = 20 (10 ND, 10 T2D), Shaded area, 95% confidence interval. r = 0.426, p = 0.061; (B) Pearson correlation between *KLF7* and *SLC2A1 (GLUT1)* expression in the same cohort. r = -0.107, p = 0.653; (C) Pearson correlation between *KLF7* and *SLC2A3 (GLUT3)* expression in the same cohort. r = 0.184, p = 0.437; (D) Pearson correlation between *Klf7* and *Slc2a2 (Glut2)* expression in mouse islets from WT, db/+, and db/db mice (GSE107489). n = 3 per group, r = 0.853, p = 0.0034; (E) Pearson correlation between *Klf7* and *Slc2a1 (Glut1)* expression in the same cohort. r = -0.835, p = 0.0051; (F) Pearson correlation between *Klf7* and *Slc2a3 (Glut3)* expression in the same cohort. r = 0.669, p = 0.0489.

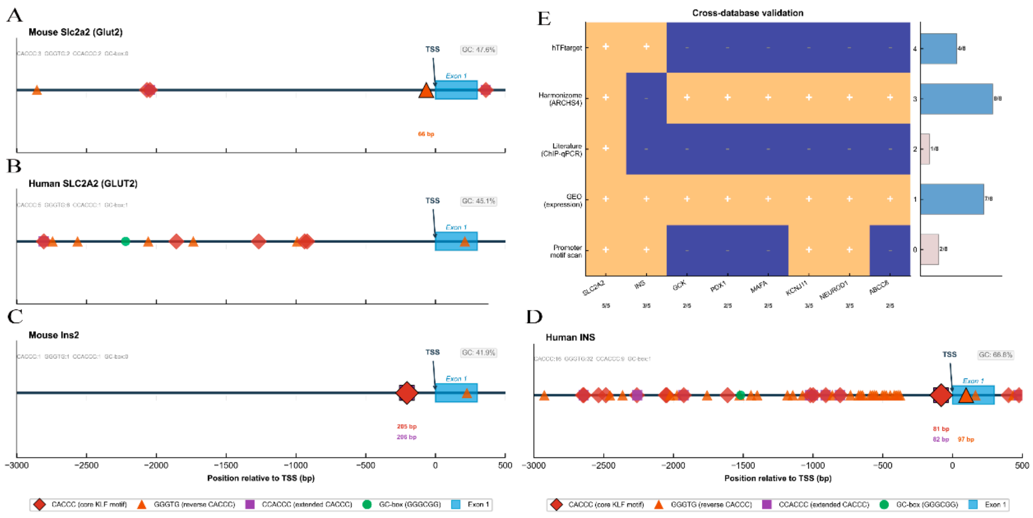

Figure S6. Bioinformatic prediction of *Klf7* binding sites in *Glut2* and *Ins2* promoters. (A) Schematic of *Klf7* TFBS distribution in the mouse *Glut2* promoter (−3,000 to +500 bp relative to TSS). CACCC (red), GGGTG (blue), CCACCC (purple), and GC-box (green) motifs are indicated; (B) KLF7 TFBS distribution in the human *GLUT2* promoter; (C) *Klf7* TFBS distribution in the mouse *Ins2* promoter; (D) *KLF7* TFBS distribution in the human *INS* promoter. Note the striking enrichment of CACCC and GGGTG motifs (58 total sites) in the human *INS* promoter, including a CCACCC box at −82 bp within the core promoter; (E) Cross-database validation matrix summarising *Klf7*/*KLF7* target gene predictions for *Glut2*/*SLC2A2* and *Ins2*/*INS* across multiple independent databases and prediction algorithms.
